# Gene networks underlying the early regulation of *Paraburkholderia phytofirmans* PsJN induced systemic resistance in Arabidopsis

**DOI:** 10.1371/journal.pone.0221358

**Published:** 2019-08-22

**Authors:** Tania Timmermann, María Josefina Poupin, Andrea Vega, Cristóbal Urrutia, Gonzalo A. Ruz, Bernardo González

**Affiliations:** 1 Laboratorio de Bioingeniería, Facultad de Ingeniería y Ciencias, Universidad Adolfo Ibáñez, Santiago, Chile; 2 Center of Applied Ecology and Sustainability (CAPES), Santiago, Chile; 3 Millennium Institute for Integrative Biology (iBio), Santiago, Chile; 4 Departamento de Genética Molecular y Microbiología, Pontificia Universidad Católica de Chile, Santiago, Chile; Academia Sinica, TAIWAN

## Abstract

Plant defense responses to biotic stresses are complex biological processes, all governed by sophisticated molecular regulations. Induced systemic resistance (ISR) is one of these defense mechanisms where beneficial bacteria or fungi prime plants to resist pathogens or pest attacks. In ISR, the defense arsenal in plants remains dormant and it is only triggered by an infection, allowing a better allocation of plant resources. Our group recently described that the well-known beneficial bacterium *Paraburkholderia phytofirmans* PsJN is able to induce *Arabidopsis thaliana* resistance to *Pseudomonas syringae* pv. tomato (*Pst*) DC3000 through ISR, and that ethylene, jasmonate and salicylic acid are involved in this protection. Nevertheless, the molecular networks governing this beneficial interaction remain unknown. To tackle this issue, we analyzed the temporal changes in the transcriptome of PsJN-inoculated plants before and after being infected with *Pst* DC3000. These data were used to perform a gene network analysis to identify highly connected transcription factors. Before the pathogen challenge, the strain PsJN regulated 405 genes (corresponding to 1.8% of the analyzed genome). PsJN-inoculated plants presented a faster and stronger transcriptional response at 1-hour post infection (hpi) compared with the non-inoculated plants, which presented the highest transcriptional changes at 24 hpi. A principal component analysis showed that PsJN-induced plant responses to the pathogen could be differentiated from those induced by the pathogen itself. Forty-eight transcription factors were regulated by PsJN at 1 hpi, and a system biology analysis revealed a network with four clusters. Within these clusters LHY, WRKY28, MYB31 and RRTF1 are highly connected transcription factors, which could act as hub regulators in this interaction. Concordantly with our previous results, these clusters are related to jasmonate, ethylene, salicylic, acid and ROS pathways. These results indicate that a rapid and specific response of PsJN-inoculated plants to the virulent DC3000 strain could be the pivotal element in the protection mechanism.

## Introduction

Plants face a myriad of pathogens and pests in their natural environment resulting in tremendous annual crop losses worldwide, ranging from 20 to 40% of potential crop yield [1,2]. Additionally, the cost of finding and developing new agrochemicals has risen from US$184 to US$286 million since 2000 [3]. Thus, in the context of a growing human population, elucidating the mechanisms of plant defense has become extremely important. Plant defense responses to biotic stresses are complex biological processes, involving numerous changes at the biochemical, physiological, and molecular (transcriptional) level, all governed by an intricate grid of hierarchical and regulatory interactions [4]. These defense mechanisms are triggered partly by the hormones salicylic acid (SA), jasmonic acid (JA), and ethylene (ET) [5–10]. Typically, SA is involved in resistance against biotrophic pathogens [11], while JA and ET participate in the regulation of defense response against necrotrophic pathogens and insects [12,13].

The induced systemic resistance (ISR) is one of these sophisticated defense strategies that is elicited by selected beneficial microorganisms, i.e., some plant growth-promoting rhizobacteria (PGPR) or beneficial fungi. Hence, plants that have previously interacted with these beneficial microorganisms deploy an enhanced defense response during subsequent pathogen or insect attacks, in a phenomenon known as priming [14–18]. In an ISR response, the defense arsenal in primed plants remains dormant and is only activated under a pathogen infection, allowing a better allocation of the plant’s energy budget [19]. Recently, a complex interplay between ISR and plant nutrient starvation responses, together with the regulation of the rhizosphere associated microbial components has been proposed [20,21]. Thus, a better understanding of the molecular mechanisms underlying ISR may foster the use of these beneficial microorganisms as a sustainable tool not only for plant disease management but also to cope with some nutrient related agricultural issues [22–24].

*Paraburkholderia phytofirmans* PsJN (formerly *Burkholderia*, [25]) is a PGPR which is able to produce positive effects in horticultural crops such as tomato, potato, grape [26–31], and also in switchgrass [32] and *Arabidopsis* [33–35]. This bacterium also induces physiological changes in plants, enhancing their adaptation to environmental stresses such as cold, salinity, and drought [26,27,33,36–40].

We recently reported that *P*. *phytofirmans* PsJN reduces disease susceptibility in *Arabidopsis thaliana* plants to the virulent strain *Pseudomonas syringae* pv *tomato* (*Pst*) DC3000, through the activation of ISR, by means of SA, JA and ET dependent signaling pathways [41]. Thus, it is possible to hypothesize that specific gene regulatory networks, associated with these hormonal signaling pathways, underlie plant protection induced by strain PsJN. Nevertheless, the molecular networks that govern ISR and specifically, the primed state of *Arabidopsis* plants colonized by *P*. *phytofirmans* PsJN are still unknown. To tackle this issue we analyzed the temporal changes in global gene expression during *Pst* DC3000 infection in *A*. *thaliana* plants that were inoculated or non-inoculated by strain PsJN. This information was used to develop a gene network analysis, which allowed identification of key transcription factors (TFs) that may be controlling the ISR response in this study model.

## Materials and methods

### Plant growth conditions and bacterial inoculation

*P*. *phytofirmans* PsJN (obtained from our laboratory stock) was grown in minimal saline medium [42], containing 10 mM fructose, in an orbital shaker (120 rpm) at 30°C. Cell suspensions were subsequently collected and adjusted to 1x10^8^ colony forming units per milliliter (CFU/ml), as determined by plate counting. Col-0 *Arabidopsis thaliana* seeds were obtained from the Arabidopsis Biological Resource Center (ABRC). The seeds were surface sterilized with 50% sodium hypochlorite (100% commercial laundry bleach) containing 0.1% Tween 20, rinsed four times with sterile water, and kept at 4°C for four days to promote uniform germination. To prepare the inoculated square Petri dishes for gnotobiotic assays, the initial inoculum of strain PsJN (1x10^8^ CFU/ml) was homogenously diluted in half strength Murashige and Skoog medium (50% MS) 0.8% agar [43] just before gelling, to reach a final inoculum of 1x10^4^ CFU/ml of medium, as this concentration was previously reported by Poupin *et al*. [34] as the optimum to induce positive effects in *A*. *thaliana* plants. Then, eight sterilized and synchronized seeds were sown per plate, in plates that were inoculated or non-inoculated with the strain, and placed vertically in a growth chamber at 22°C with a photoperiod of 16/8 h (light/dark).

### Infection of plants and RNA isolation

**Fig 1:**
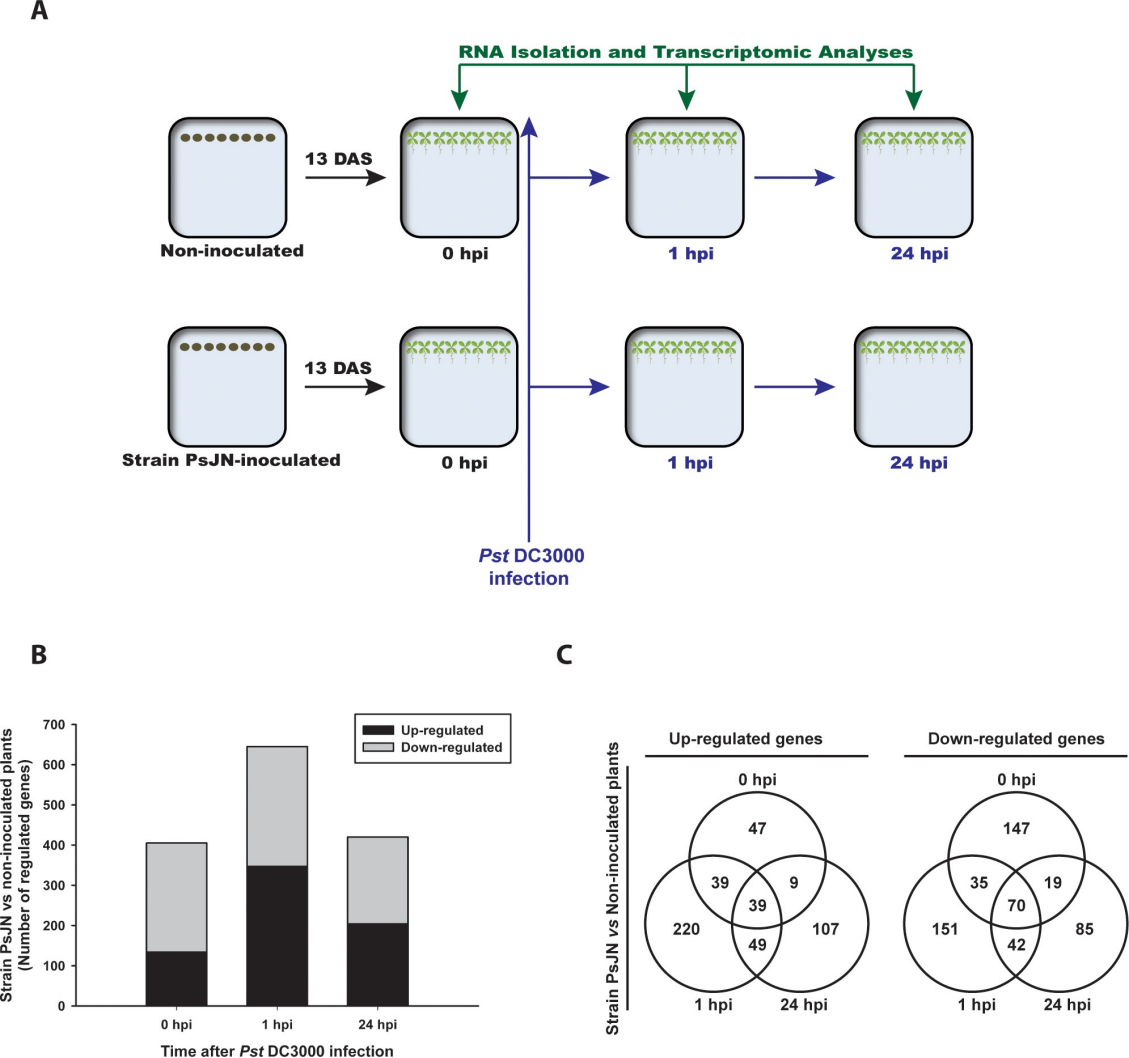
Transcriptomic changes induced by *Paraburkholderia phytofirmans* PsJN in *Arabidopsis thaliana* plants infected with *Pseudomonas syringae* DC3000. (A) Graphical explanation of the experimental design where *A*. *thaliana* seeds were inoculated (strain PsJN-inoculated) or non-inoculated at sowing, and 13 days after that (DAS) a group of plants were sampled and analyzed (0 hours post infection, hpi) and the remaining plants were infected with *P*. *syringae pv*. *tomato* DC3000, to collect and analyze samples at 1 and 24 hpi (B) Number of *A*. *thaliana* genes that were either up- or down-regulated by *P*. *phytofirmans* PsJN before (0 hpi) and after *P*. *syringae* DC3000 infection (1 and 24 hpi) in comparison to non-inoculated plants (Rank-product, adjusted p<0.05) (C) Venn diagrams of the data presented in (B) showing the overlap of genes that were up- or down-regulated in *Arabidopsis* plants inoculated with strain PsJN before (0 hpi) and after the infection with *Pst* DC3000 (1 and 24 hpi).

A graphical representation of the experimental design is presented in Fig 1A. Briefly, *Pst* DC3000 (obtained from Loreto Holuigue’s Laboratory) was grown on King’s B medium in an orbital shaker (120 rpm) at 28°C, supplemented with 50 μg/ml of rifampicin and 50 μg/ml of kanamycin, as selection antibiotics. Thirteen days after sowing (13 DAS), *A*. *thaliana* plants with four visible leaves (LP.04 stage [44]), inoculated or non-inoculated with *P*. *phytofirmans* PsJN, were sampled and stored in 1.5 ml Eppendorf tubes containing RNAlater (Ambion, Austin, TX) according to the manufacturer's instructions. These treatments correspond to 0 hours post-infection (0 hpi). Then, in the remaining plants, rosettes were infected by spraying 100 μl per plate of *Pst* DC3000 at 1x10^5^ CFU/ml in 10 mM MgCl_2_ (Fig 1A). One and 24 h after *Pst* DC3000 infection (1 and 24 hpi), five pools with four plants each were collected per treatment (non-inoculated plants/*Pst* and strain PsJN-inoculated plants/*Pst*) and stored in RNAlater (Ambion, Austin, TX) until RNA extraction (according to the manufacturer's instructions). For RNA extraction, plants were ground with an electronic pestle in the Eppendorf tube without the RNAlater solution. Then, the total RNA of each sample was extracted using the Trizol reagent (Invitrogen, Carlsbad, CA) according to the manufacturer’s recommendations.

### Microarray hybridization

Three biological replicates, consisting of four plantlets of 13 DAS each, non-inoculated or PsJN-inoculated treatments, infected or not with *Pst* DC3000, were used for global gene expression analysis using the GeneChip *Arabidopsis* ATH1 Genome Array (Affymetrix, Santa Clara, CA). RNA samples were quantified and analyzed using a NanoDrop (Thermo Scientific, USA) spectrophotometer, according to the manufacturer’s instructions. For RNA processing, we used the GeneChip 3’ IVT Express Kit (Affymetrix, Santa Clara, CA), following the manufacturer’s instructions. First-stranded cDNA synthesis was achieved with 0.5 μg of RNA from each sample, using T7 oligo(dT) Primer and the First-strand enzyme mix (Affymetrix, Santa Clara, CA). Subsequently, IVT biotin-labeled aRNA (cRNA) was generated using the second-stranded cDNA as a template and following the manufacturer’s specifications. Later, the biotin-labeled aRNAs were fragmented (between 35 and 200 bases in length) and hybridized on a GeneChip *Arabidopsis* ATH1 Genome Array using standard procedures (45°C for 16 h). The arrays were finally washed and stained in a Fluidics Station 450 (Affymetrix, Santa Clara, CA).

### Affymetrix data processing and analysis

ATH1 arrays were scanned with the GeneChip scanner 300 and the images were analyzed using the GeneChip Operating Software. GeneChip arrays data were first evaluated using a set of standard quality control steps described by the manufacturer. Raw data were normalized by RMA (Robust Multi-Array Average) [45] using the *affy* R package [46]. RMA expression values (log2-transformed) for each set of biological replicates were evaluated using Pearson correlation and ranged from 0.96 to 0.99. The data analyzed in this work were deposited in NCBI's Gene Expression Omnibus (GSE124475) [47]. Differentially expressed genes (DEGs) in response to *Pst* DC3000 infection influenced by strain PsJN inoculation were determined in normalized data using a two-way analysis of variance (ANOVA) with an adjusted *p*<0.01 by Benjamini, and Hochberg false discovery rate correction [48] by the Multiple Experiment Viewer (MEV) software [49]. We used a multiple regression with expression Y of a given gene i calculated as Yi = β0 + β1Pst + β2PsJN + β3Pst/PsJN + ε, where β0 is the global mean, and β1, β2, and β3 are the effects of the *Pst* DC3000 infection, PsJN inoculation and the interaction between these two factors, respectively. The variable ε corresponds to the unexplained variance (noise). To identify DEGs between two experimental conditions (e.g., non-inoculated and strain PsJN-inoculated at one hpi *Pst* DC3000-infection), we used the Rank-Product analysis (*p*<0.05) in the group of selected DEGs by ANOVA [50]. We also performed a principal component analysis (PCA) using Log2 normalized expression data across each sample, as described before [51], and two-dimensional coordinates were plotted using MEV software. For functional analysis, the Virtual-Plant [52] and Revigo [53] platforms were used. For those Affymetrix IDs that represent more than one *locus*, all of them were considered for further functional analyses. Gene Ontology terms (GO) that were statistically overrepresented were calculated using the BioMaps tool in the Virtual-Plant platform (Fisher Exact Test, *p*< 0.01 with FDR correction). For graph purposes, the REVIGO platform was used to reduce semantically redundant GO terms [53]. Additionally, MapMan software [54] was used to provide a graphic representation of DEGs involved in the biotic stress metabolic pathway before and after the infection with *Pst* DC3000.

### TFs identification and Gene Network analysis

To identify the regulated genes corresponding to TFs encoding genes, we selected the group of DEGs in the treatment with the largest transcriptional changes, corresponding to plants at 1 hpi with *Pst* DC3000, where 645 DEGs were up- and down-regulated by strain PsJN. Then, this list of genes was intersected, using the VirtualPlant platform [52], with a list of 2273 TFs obtained from Jin et al. [55] and Pérez-Rodríguez et al. [56]. Gene families were assigned using the TAIR platform (https://www.arabidopsis.org).

For Gene Network analysis, we also selected the group of DEGs identified by Rankproduct between non-inoculated and inoculated with strain PsJN at 1 hour after the infection with *Pst* DC3000 (*p*<0.05). We included the following information: (1) a gene co-expression network from the ATTED-II database [57], where the co-expression values were calculated using weighted Pearson’s correlation coefficient, as described by Obayashi et al. [58]; (2) the protein-DNA interactions described in DAP-seq database [59], where we filtered by the over-representation of the TF binding site in the upstream gene region (1000 bp) (two standard deviations) above the mean occurrence in all the upstream sequences in the genome, and by the TFs/target pairs for which co-expression values were significantly correlated (adjusted *p*<0.05 by FDR) in our microarray experiment. Then, we used the Cytoscape software [60] for visualizing the resulting network, where nodes represented genes and they are connected by edges that represent putative regulatory interactions. The network topology was described using a community cluster (Glay) analysis in Cluster-Maker tools [61]. This analysis recognizes functional groups within nodes and finds densely connected regions [62]. Finally, statistical overrepresentation in biological functions in each cluster was identified using a hypergeometric analysis in Clue-GO tools (adjusted *p*<0.01 by FDR) [63].

### qRT-PCR Analyses

To perform qRT-PCRs analyses, the same RNAs used for Affymetrix analyses were used to prepare cDNA from the samples collected at 1 and 24 hpi (Fig 1A); cDNA was prepared using 1 μg of total RNA (previously treated with DNAse I RQ1, Promega, USA), random hexamer primers and the Improm II reverse transcriptase (Promega, USA), according to the manufacturer’s instructions. Real time RT-PCRs were performed using the Brilliant SYBR Green QPCR Master Reagent Kit (Agilent Technologies, USA) and the Eco Real-Time PCR detection system (Illumina, USA) as described by Poupin et al. [34]. The PCR mixture (10 μl) contained 4.75 μl of template cDNA (diluted 1:10) and 140 nM of each primer. Amplifications were performed under the following conditions: 95°C for 10 min, followed by 40 cycles of 94°C, 30 s; 57–60°C, 30 s; and 72°C, 30 s, followed by a melting cycle from 55 to 95°C. Primer pairs used were described in [64] and in [34], standard quantification curves with serial dilutions of PCR products were constructed for each amplicon to calculate the amplification efficiency (E) of each primer set, according to the equation: E = 10^(− 1/ slope)^ − 1 [65]. Gene expression levels were calibrated for each time (1 or 24 hpi) using the average value in the samples of non-inoculated plants. *TIP4* was used as a housekeeping (HK) gene since it showed a high stability with a standard deviation of 0.6 (n = 30). Then, an accurate ratio between the gene of interest (GOI) and the HK gene expression was calculated for each sample, using the equation:

$$\frac{1+{E(GOI)}^{-(ctGOI-ctGOI\;calibrated)}}{1+{E(HK)}^{-(ctHK-ctHK\;calibrated)}}$$

Efficiency values for each primer set were close to 1 and R^2^ values were over 99% [66]. All experiments were performed in three biological (four plants each), and two technical replicates.

## Results

### Transcriptional changes regulated by *P*. *phytofirmans* PsJN during systemic resistance to a virulent infection in *Arabidopsis*

To get a better understanding of the molecular changes underlying the ISR triggered by strain PsJN in *Arabidopsis* plants infected with a virulent bacterium, a transcriptome-profiling assay using non-inoculated plants and strain PsJN-inoculated (0 hpi) plants was performed. Additionally, the analyses included samples 1 and 24 h after a challenge with *P*. *syringae* pv. tomato DC3000 (1 and 24 hpi) (Fig 1A). The complete list of genes regulated by strain PsJN in the different treatments and times is presented in S1 Table. To first analyze the transcriptional changes induced only by strain PsJN inoculation, the regulated genes were compared between strain PsJN-inoculated and non-inoculated plants (0 hpi). The presence of strain PsJN significantly up-regulated 134 genes and down-regulated 271 genes (Rank-Product method; *p*<0.05) (Fig 1B; 0 hpi). Transcriptional changes were transiently intensified at 1 h after the challenge with *Pst* DC3000 (Fig 1B, 1 hpi), where 347 genes were significantly up-regulated and 298 down-regulated by the previous inoculation with strain PsJN. Finally, at 24 h after *Pst* DC3000 infection, 204 genes were significantly up-regulated and 216 down-regulated by the PGPR (Fig 1B).

To analyze gene co-regulation by the different treatments, the list of DEGs were compared as shown in Fig 1C. Only 22.5% of the genes that were up-regulated in strain PsJN-inoculated plants at 1 hpi were also up-regulated before the infection; 25.4% of the up-regulated genes at 1 hpi remained in that condition at 24 hpi, and 39 genes were up-regulated in all the measured times, independently of the infection and seem to be related exclusively to the presence of strain PsJN (Fig 1C). Regarding the down-regulated genes, 70 genes were affected in all the measured times and could be associated with a specific response to strain PsJN, corresponding to 32.4% of the reaction triggered at 24 hpi (Fig 1C).

Since the largest transcriptional changes were observed at 1 hpi in inoculated plants, a gene ontology (GO) term enrichment analysis (*p*<0.01) was performed at this time using the BioMaps tool, in the VirtualPlant platform [52]. To simplify interpretation and avoid redundancy, significant GO term lists were reduced using REVIGO tools, but full lists of all significant GO terms with the group of DEGs included in each term are presented in S2 Table. Thus, the GO terms in the group of up-regulated genes that were statistically overrepresented in comparison with those represented in the *Arabidopsis* genome arrays were “Response to stimulus”, “Response to stress”, “Response to biotic stimulus”, “Response to oxidative stress”, and “Response to other organisms” (S1A Fig). Likewise, genes implicated in “cellular response to nutrient levels” were predominantly up regulated in strain PsJN-inoculated plants at 1 hpi, suggesting a better use of plant nutrients or energy. For genes that were down-regulated, the most significantly enriched GO terms in biological process were “Response to stimulus”, “Response to stress”, “Response to abiotic stimulus”, “Response to organic substance”, “Response to hormone”, and “Secondary metabolic process” (S1B Fig).

**Fig 2:**
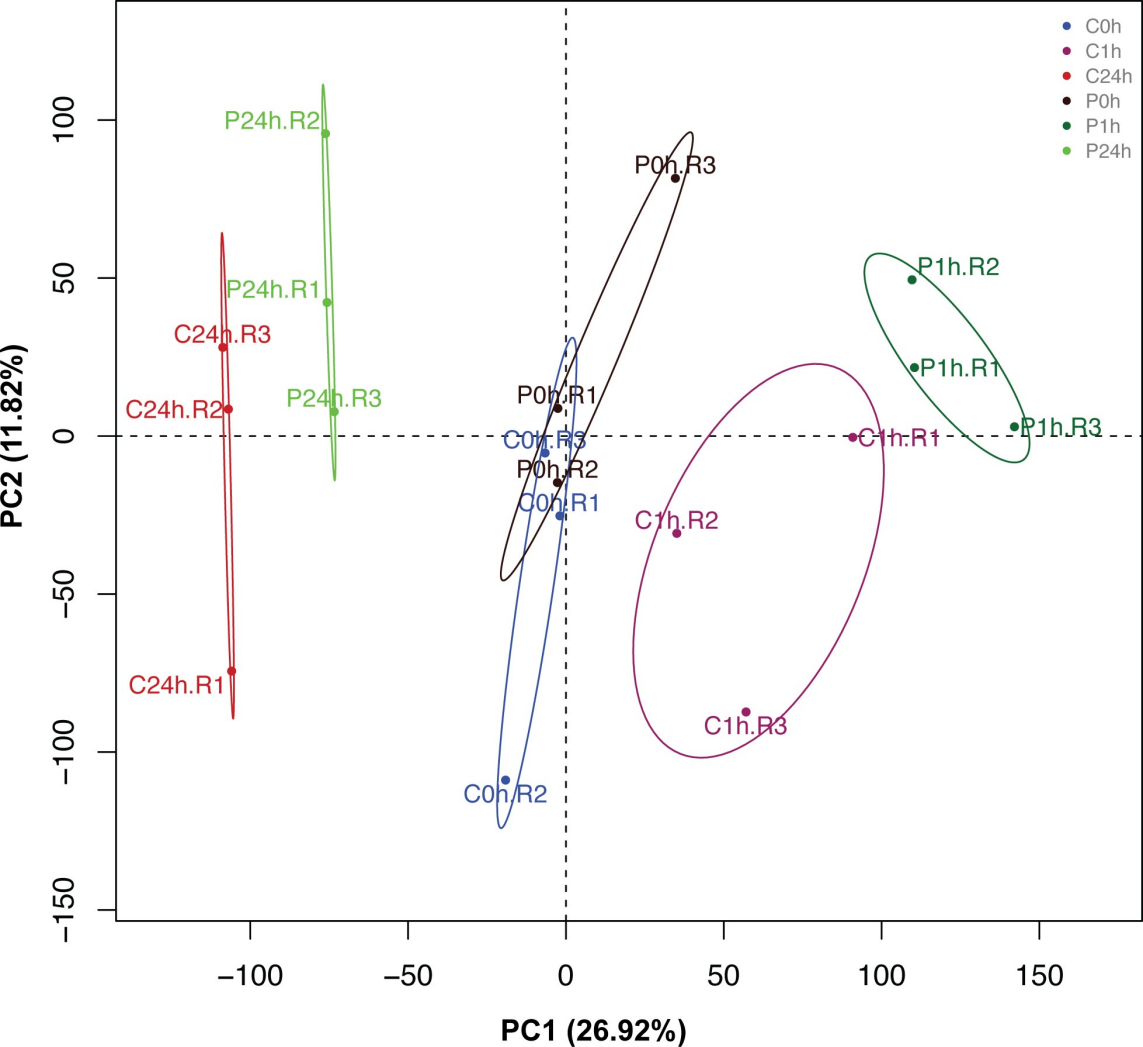
Principal component analysis (PCA) of global gene expression data in *Arabidopsis* plants inoculated or non-inoculated with *Paraburkholderia phytofirmans* PsJN before and after the challenge with *Pseudomonas syringae* DC3000. Colored dots denote each biological replicate. “C” and “P” indicate non-inoculated plants and *P*. *phytofirmans* PsJN-inoculated plants, respectively, while the number next to each dot indicates the sampling time. R indicates the replicate number of the Affymetrix chip.

A PCA (Fig 2) showed that the first principal component (PC1) accounted for 26.9% of the total variation and grouped the samples based on time (0 h, 1 and 24 hpi), regardless of the infection with *Pst* DC3000. On the other hand, PC1 also allows the differentiation between non-inoculated and strain PsJN-inoculated plants at 1 hpi, which is in agreement with the highest transcriptional changes observed at this time (Fig 1B and 1C). The second principal component (PC2) accounted for 11.8% of the variation, and differentiated strain PsJN-inoculated and non-inoculated plant samples in all the measured times.

**Fig 3:**
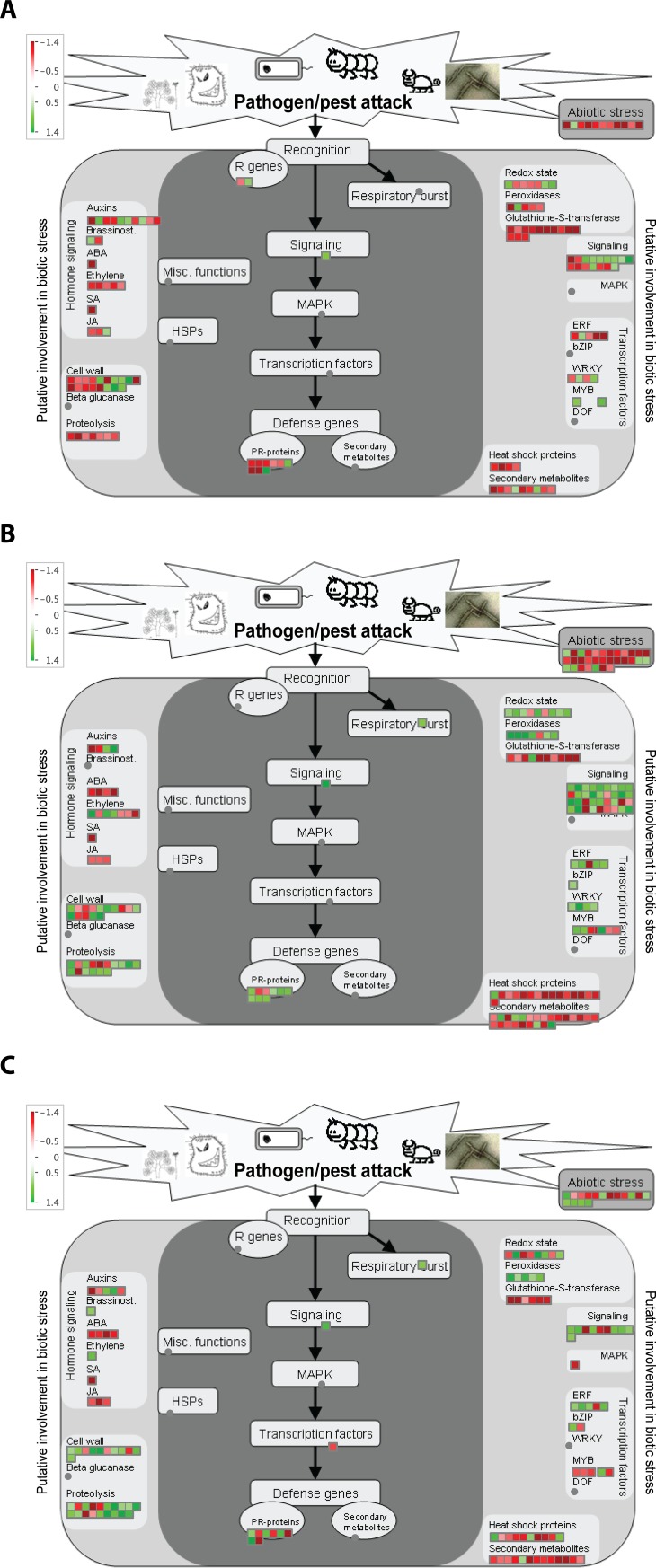
Biological processes affected by *Paraburkholderia phytofirmans* PsJN inoculation in *Arabidopsis* plants before and after the challenge with *Pseudomonas syringae* DC3000. MapMan software visualization of the defense functions affected in response to *P*. *phytofirmans* PsJN in *A*. *thaliana* plants before the challenge with *P*. *syringae* DC3000 (A); at 1-hour post infection (B) or at 24 hours post infection (C). Differential expressed genes were binned to MapMan functional categories with regard to pathogen/pest attack and Log2 fold changes values for each gene are represented. Up-regulated and down-regulated transcripts are shown in green and red, respectively.

The MapMan software [54] was utilized to have an overview of the pathways related to biotic stress which were affected by strain PsJN inoculation before *Pst* DC3000 infection and at 1 and 24 hpi. Before the pathogen infection (Fig 3A), plants inoculated with strain PsJN showed partial activity of auxin, ET, brassinosteroids (BR), and JA signaling; as well as regulation of genes related to the cell wall; redox state; peroxidases, signaling, PR proteins and ERF, WRKY and MYB TFs. At one hpi, a high increase in the regulation of signaling related genes, abiotic stress, secondary metabolites, heat shock proteins and proteolysis was observed (Fig 3B). Additionally, a tendency towards an up-regulation (compared to 0 hpi) was observed in ET, redox state, peroxidases, and with the ERF, bZIP, MYB and WRKY TFs (Fig 3B). Accordingly to the previous analyses, the transcriptional changes decreased at 24 hpi in comparison to at 1 hpi (Fig 3C).

**Fig 4:**
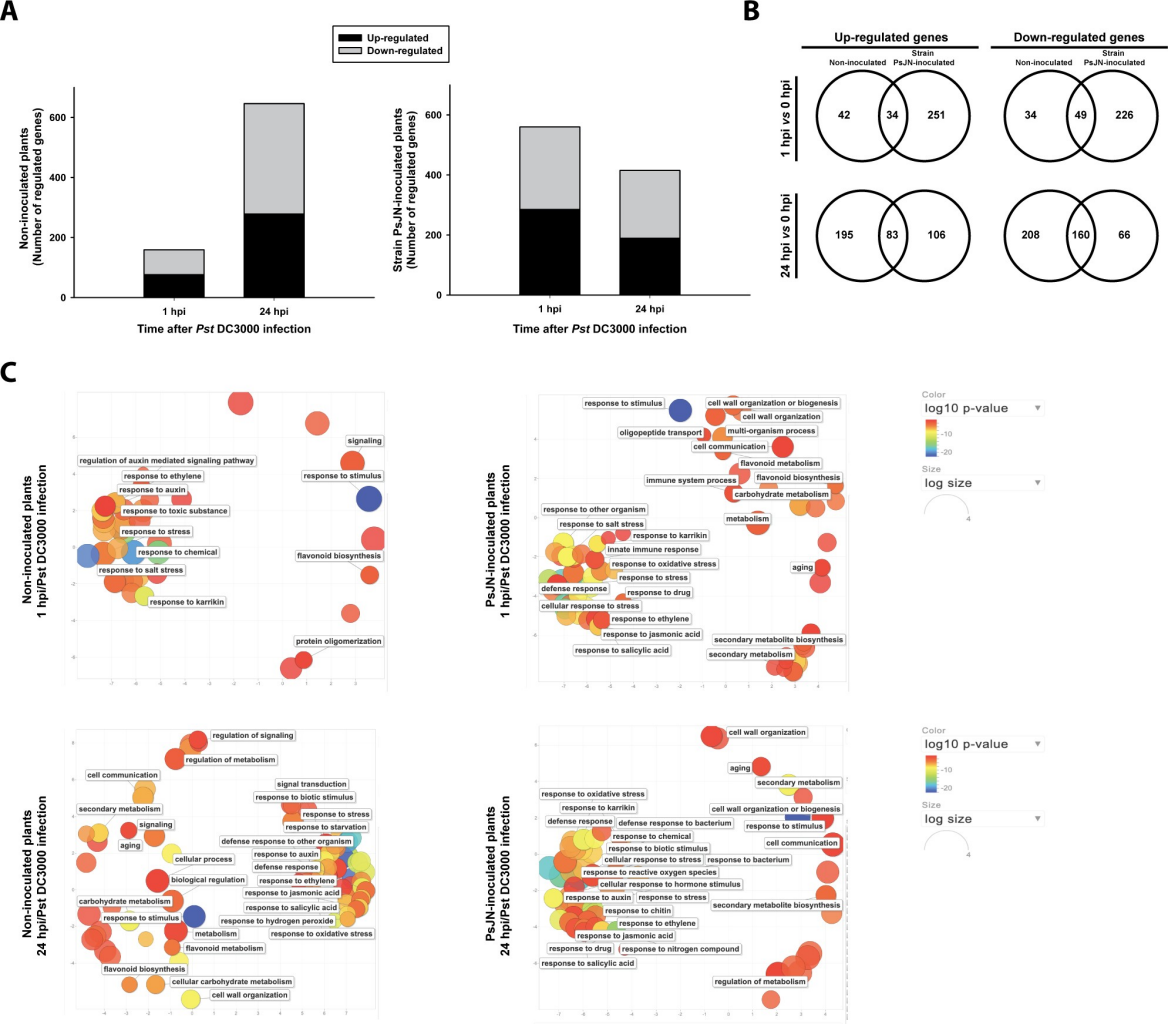
Gene regulation induced by *Pseudomonas syringae* DC3000 in *Arabidopsis* plants inoculated or non-inoculated with *Paraburkholderia phytofirmans* PsJN. Number of regulated genes at 1 and 24 hours post-infection (hpi) with *Pst* DC3000 in plants that were previously inoculated with strain PsJN (PsJN-inoculated plants, right panel in A) or were not inoculated with the same strain (Non-inoculated plants, left panel in A), all data were compared to 0 hpi (Rank-product, adjusted *p*<0.05) (B) Venn diagrams of the data presented in (A) showing the overlap of genes that were either up- or down-regulated in *Arabidopsis* plants inoculated or non-inoculated with strain PsJN at 1 and 24 hpi of *Pst* DC3000 infection. Panel (C) shows biological processes affected by *Pst* DC3000 infection in PsJN- and non-inoculated plants (right and left panel, respectively) at 1 (upper figures) or 24 hpi (bottom figures). Gene Ontologies (GO) terms significantly enriched for biological processes (*p<*0.01) are represented by circles and clustered according to semantic similarities using the Revigo tool. Circle sizes are proportional to the number of genes in each GO term, whereas the color scale indicates the Log_10_ *p-*value obtained in the enrichment analysis (red higher, blue lower).

Then, and to isolate the effects of *Pst* DC3000 infection, the transcriptional data were compared using the infection as a factor inside each group of PsJN-inoculated or non-inoculated plants (S3 Table; Fig 4). While non-inoculated plants showed a stronger response at 24 hpi than at 1 hpi (646 vs 159 genes, respectively), strain PsJN-inoculated plants responded earlier, regulating 560 genes at 1 hpi and showing a lower response magnitude at 24 hpi (415 genes) (Fig 4A). Thus, at 24 hpi, the magnitude of transcriptional changes was slightly lower (64%) in strain PsJN-inoculated plants compared with the non-inoculated ones (Fig 4A). Moreover, at one hpi, 13% of the genes regulated by strain PsJN inoculation were also regulated in non-inoculated plants and could be related only to the strain DC3000 infection; this shared response was 29.7% at 24 hpi (Fig 4B). When GO that are overrepresented are compared between the treatments, different patterns related with PsJN inoculation can be observed (Fig 4C). For instance, at 1 hpi regulation of SA and JA was only observed in PsJN inoculated plants, as well as defense response and cell wall organization (Fig 4C). A similar situation was observed at 24 hpi, were GO are qualitatively different in plants that were previously inoculated with PsJN (Fig 4C).

### TFs regulated by *P*. *phytofirmans* PsJN, and their associated networks, during ISR response to a virulent pathogen

To identify the TFs regulated in this ISR response, the DEGs at 1 hpi in inoculated plants (the treatment with major transcriptional changes) were compared with the *Arabidopsis* TFs described in Jin et al. [55] and Pérez-Rodríguez et al. [56] using the Virtual Plant platform [52]. Thirty-one TFs coding genes were identified as up-regulated, belonging to several gene families, and 17 genes were down regulated. Table 1 indicates their fold changes and *p*-values at 0, 1 and 24 hpi. Here, some gene families possessed genes both up- and down-regulated, such as, bHLH, ERF/AP2, MYB, and NAC, whereas families such as WRKY presented only up-regulated members and others only down-regulated ones (i.e. C2H2).

**Table 1:** Transcription factors coding genes regulated by *Paraburkholderia phytofirmans* PsJN at 1 hour post infection (hpi) with *Pseudomonas syringae* Pst DC3000 *in Arabidopsis thaliana plants*, and their fold changes and *p-*values before the infection (0 hpi) and at 24 hpi.

			Fold change (Log2)/*p* value		
Gene family	Locus	Name	0 hpi	1 hpi	24 hpi
**UP-REGULATED GENES**					
ARR	At1g67710	*ARR11*	NS	0.88/0.02	NS
ARR	At1g19050	*ARR7*	NS	0.78/0.04	NS
bHLH	At2g43140	*BHLH129*	NS	1.99/0	1.18/0.01
bHLH	At1g05805	*AKS2*	NS	1.07/0.01	0.71/0.05
bHLH	At5g15160	*BHLH134*, *BNQ2*	1.19/0	1.04/0.01	NS
bZIP	At5g49450	*ATBZIP1*	NS	0.78/0.05	1.25/0.01
bZIP	At1g13600	*ATBZIP58*	NS	0.77/0.04	NS
CCCH	At2g05160	*CCCH-type zinc finger family protein with RNA-binding domain-containing protein*	0.7/0.04	1/0.01	NS
ERF/AP2	At3g16770	*ATEBP*, *EBP*, *ERF72*, *RAP2*.*3*, *RELATED TO AP2 3*	0.76/0.03	0.82/0.03	NS
ERF/AP2	At4g39780	*member of the DREB subfamily A-6 of ERF/AP2 transcription factor family*	NS	1.11/0.01	NS
ERF/AP2	At3g60490	*member of the DREB subfamily A-4 of ERF/AP2 transcription factor family*	NS	1.04/0.01	NS
ERF/AP2	At1g06160	*ERF59*, *ORA59*	NS	0.98/0.02	NS
ERF/AP2	At1g36060	*WIND3*	NS	0.9/0.04	NS
GARP	At1g13300	*TNIGT1*, *HRS1*, *NIGT1*.*4*	NS	0.87/0.02	NS
GATA	At2g45050	*GGATA2*	NS	0.79/0.03	NS
KANADI	At5g42630	*KAN4*, *KANADI 4*	NS	0.92/0.02	NS
**MYB**	**At1g74650**	***ATMYB31***	**NS**	**1.1/0.01**	**NS**
MYB-like	At5g06800	*myb-like HTH transcriptional regulator family protein*	NS	0.8/0.04	NS
NAC	At3g04070	*ANAC047*	NS	2.16/0	NS
NAC	At1g69490	*ANAC029*	0.83/0.03	1.02/0.02	NS
NAC	At3g15500	*ANAC055*, *ATNAC3*	NS	0.85/0.04	NS
PLATZ	At1g76590	*PLATZ transcription factor family protein*	NS	1.2/0.01	NS
WRKY	At5g07100	*ATWRKY26*	NS	1.51/0	NS
**WRKY**	**At4g18170**	***ATWRKY28***	**NS**	**0.94/0.04**	**NS**
WRKY	At5g52830	*ATWRKY27*	NS	0.79/0.04	NS
WRKY	At4g01720	*ATWRKY47*	NS	0.78/0.05	NS
U	At1g32540	*LOL1*	1.14/0	1.08/0.01	0.88/0.22
U	At5g02810	*APRR7*	NS	0.91/0.02	NS
U	At2g19810	*ATOZF1*, *ATTZF2*	NS	0.8/0.04	NS
U	At4g29190	*ATOZF2*, *TZF3*	NS	0.76/0.04	NS
U	At4g29080	*IAA27*, *PAP2*	NS	0.69/0.05	NS
**DOWN-REGULATED GENES**					
B-BOX ZINC FINGER	At2g21320	*BBX18*	NS	-0.78/0.04	NS
bHLH	At5g04150	*BHLH101*	-3.13/0	-3.73/0	-2.77/0
bHLH	At3g56980	*BHLH039*, *ORG3*	-3.31/0	-3.96/0	-3.26/0
C2H2	At5g59820	*ATZAT12*, *RHL41*, *ZAT12*	NS	-0.89–0.02	NS
CCAAT-HAP5	At1g56170	*ATHAP5B*, *HAP5B*, *NF-YC2*	NS	-0.9/0.02	-1.12/0.01
ERF/AP2	At5g25190	*ESE3*	NS	-0.79/0.04	NS
**ERF/AP2**	**At4g34410**	***ERF109*, *RRTF1***	**NS**	**-0.8/0.03**	**NS**
ERF/AP2	At1g74930	*ORA47*	NS	-1.51/0	NS
HSF	At2g26150	*ATHSFA2*	NS	-1.35/0.01	NS
MYB	At5g07690	*ATMYB29*, *PMG2*, *RAO7*	NS	-1.09/0.01	NS
MYB	At1g18710	*ATMYB47*	NS	-1.34/0	NS
MYB-like	At1g01520	*ASG4*, *REV3*	NS	-0.83/0.03	-1.06/0.02
**MYB-related**	**At1g01060**	***LHY*, *LHY1***	**NS**	**-0.91/0.02**	**NS**
NAC	At1g01720	*ANAC002*, *ATAF1*	NS	-0.95/0.01	-1.24/0.01
NAC	At5g63790	*ANAC102*	-0.94/0.03	-1.02/0.01	-1.23/0.01
NAC	At1g77450	*ANAC032*	-1.67/0	-1.74/0	NS
U	At1g19180	*ATJAZ1*, *TIFY10A*	NS	-0.77/0.04	NS

U: Undetermined (gene families were assigned using TAIR). NS: Non-statistically significant. Genes in bold are those identified as central hubs in network analyses. Families marked with the same color have members both up- and down-regulated.

**Fig 5:**
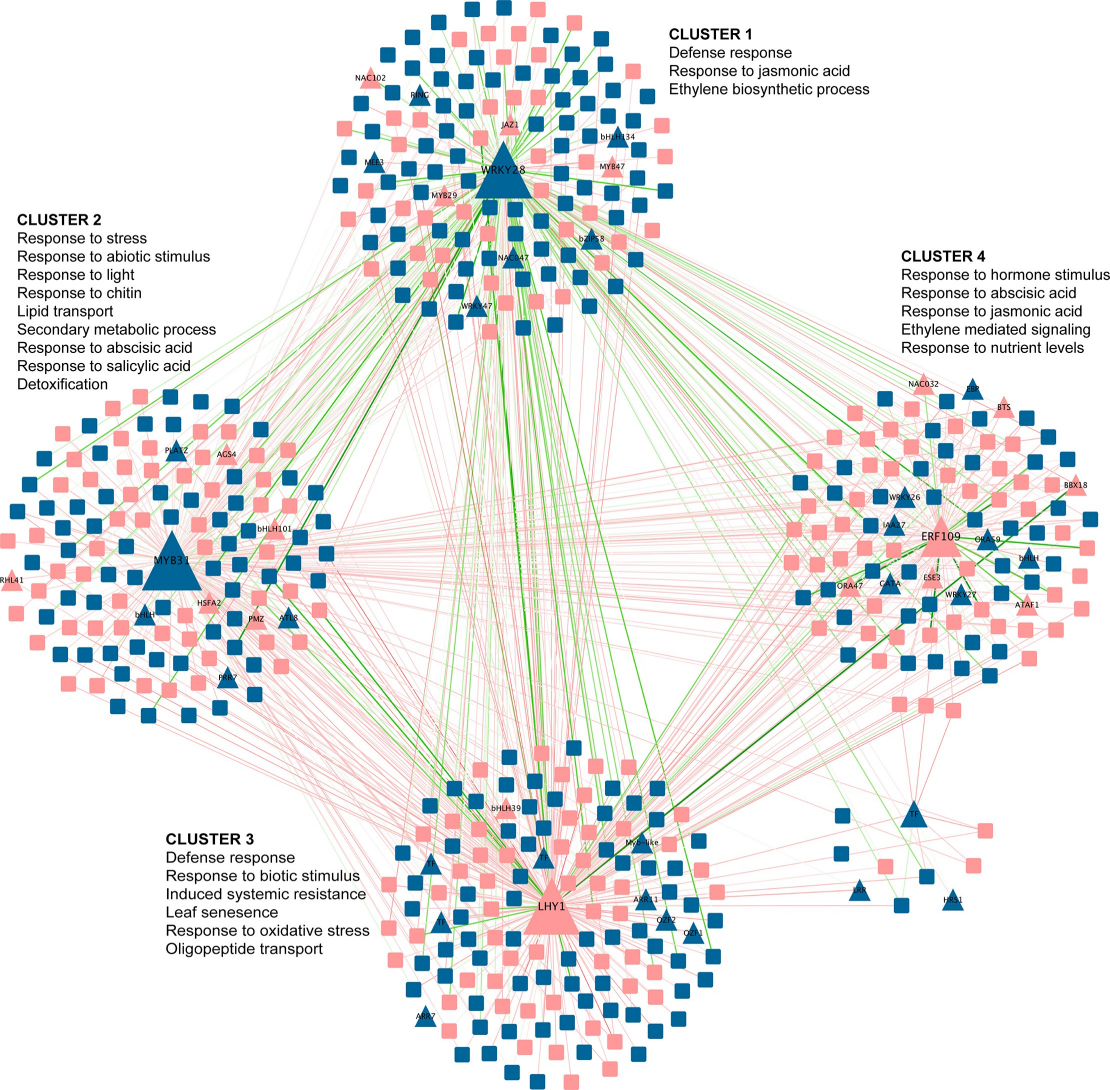
*Pseudomonas syringae* DC3000-responsive regulatory gene network influenced by the inoculation with *Paraburkholderia phytofirmans* PsJN 1 hour post infection (hpi). Network analysis of genes with significant differential expression due to inoculation with strain PsJN at 1 hpi with *Pst* DC3000 (*p*<0.05 corrected by FDR). Genes are presented as triangles (transcription factors, TFs) and squares (target genes). Colors are used to distinguish up-regulated (blue) and down-regulated (pink) genes. The size of triangle represents the number of putative connections (target genes). Clustering analysis grouped by topology the four TFs most connected (LHY1, WRKY28, MYB31 and Redox-responsive TF). Statistically overrepresented biological functions of the four modules identified in cluster analysis are presented next to each cluster. Lines indicate predicted positive (green) or negative (red) co-expression regulation.

Then, a network inference analysis was performed with DEGs in PsJN-inoculated plants at 1 hpi. We included protein-DNA interactions based on the DAP-seq database [59] and a gene co-expression database [58]. As shown in Fig 5, a network comprising 500 genes with 975 interactions was generated with the DEGs, showing their putative regulatory interactions. Genes were represented as nodes that are connected by edges based on their regulatory interactions. We identified four co-expression clusters using the community cluster (Glay) algorithm in ClusterMaker tools [61; 62]. LHY1, WRKY28, MYB31 and a Redox-responsive TF (RRTF1/ERF109) were the most connected and central TFs within each co-expression cluster (Fig 5). Additionally, inside each module we found several TFs and other signaling components. In addition, statistically overrepresented biological functions in the four modules using a hypergeometric test in Clue-GO tools (adjusted p<0.05 by FDR) [63] were described. Cluster 1, containing 130 genes putatively regulated by WRK28, was statistically enriched in genes associated with ethylene biosynthesis process, response to JA and defense response. Consistent with this functional analysis, MYB47, ANAC102 and ANAC047 were comprised within this module. These TFs have been shown to be involved in JA/ET signaling pathways [67–69]. Therefore, this module also includes the TFs MYB29 and WRKY47, associated with stress and defense response, respectively [70,71]. Remarkably, the network includes important functions for plant defense against pathogens, such as the responses to chitin and reactive oxygen species (clusters 2 and 3). Moreover, genes related to abscisic acid (ABA) and SA stimulus, ET signaling, light response and detoxification were also identified as overrepresented in these modules.

**Fig 6:**
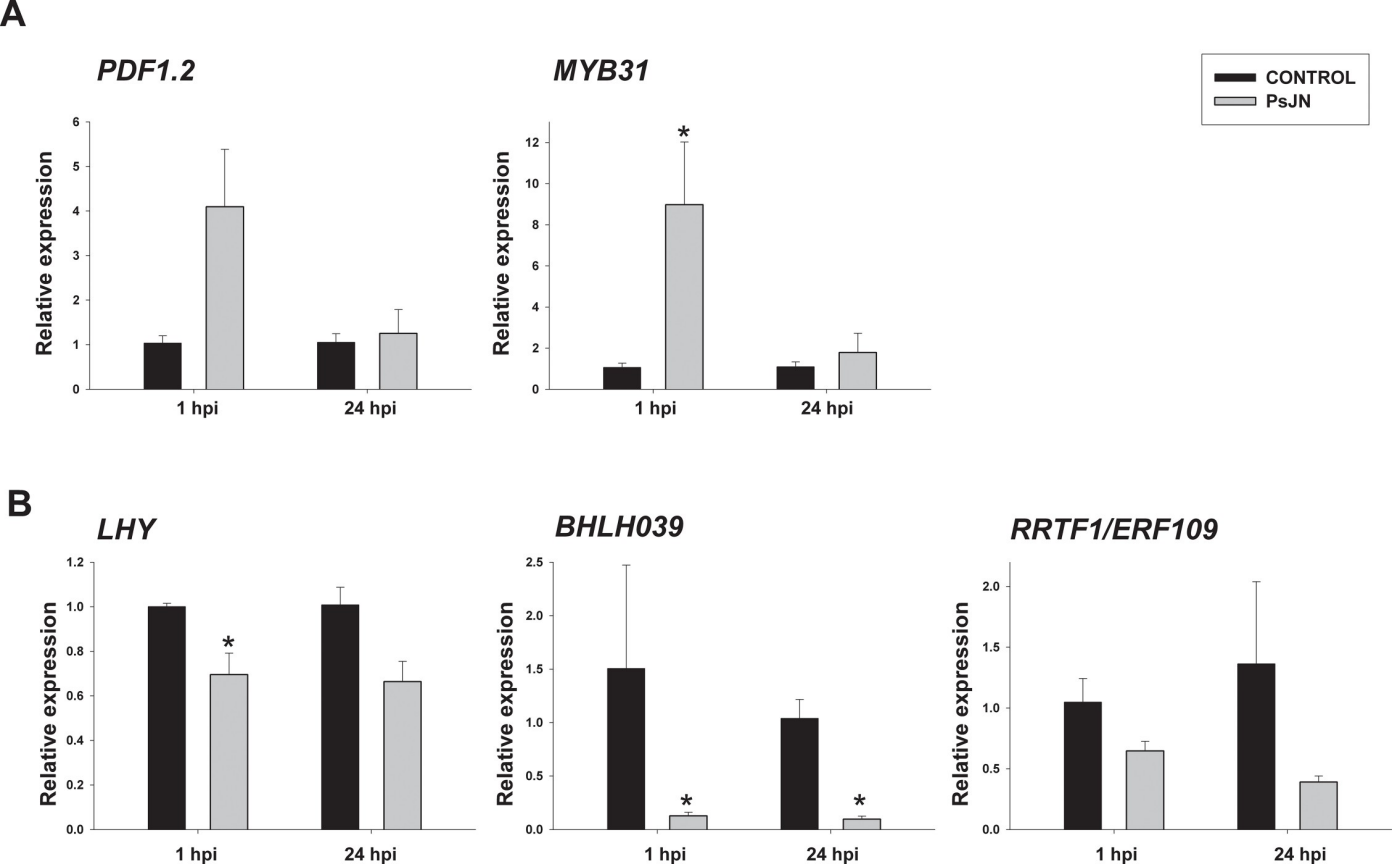
Temporal quantitative real-time PCR of selected transcription factors-coding genes and *PDF1*.*2* in *Arabidopsis* in response to *Paraburkholderia phytofirmans* PsJN inoculation after a challenge with *Pseudomonas syringae* DC3000 strain. Relative gene expression of selected genes at 1 and 24 hours post infection (hpi) with strain Pst DC3000 in plants that were inoculated (gray bars) or not (control, black bars) with strain PsJN. Genes with an up-regulated pattern at 1 hpi are shown in (A). Those with a down-regulated pattern at 1 hpi are shown in (B). Data are means ± SE of three biological replicates per treatment and two technical replicates. Normalization was performed with the housekeeping *TIP4* gene. Asterisk indicates statistical significance among treatments in a particular time (One way ANOVA, *p*<0.05).

The same RNAs used for the transcriptome-profiling assay were used to analyze the expression of five TFs by qRT-PCR, and their expression patterns were studied at 1 and 24 hpi (Fig 6). Similar expression patterns were obtained in comparison with the data obtained by Affymetrix (Table 1), except with *WRKY28*, which was not detected by qRT-PCR. Here *MYB31* was up regulated by PsJN at 1 hpi and non-regulated at 24 hpi (Fig 6A). *LHY*, *BHLH039* and *RRTF1/ERF109* showed a down-regulation pattern at 1 hpi (Fig 6B). Concordantly with the Affymetrix analysis, *BHLH039* remained down regulated at 24 hpi (Fig 6B). Additionally, the expression pattern of *PDF1*.*2* (*PLANT DEFENSIN 1*.*2*) was also analyzed, as a marker that has been associated to PsJN inoculation in our previous studies [34, 41], finding an up-regulation (not-statistically significant) at 1 hpi (Fig 6A).

## Discussion

### A fast and specific molecular response support strain PsJN-induced resistance to *P*. *syringae* DC3000

It is well documented that some PGPR can increase plant resistance to several pathogens through ISR, in a manner that is highly dependent on the combination of the particular PGPR strain, the plant species and even the pathogen [13,72]. However, the molecular mechanisms underlying these complex inter-kingdom interactions are far from being elucidated. Some studies have reported transcriptomic changes in plants after the inoculation with a beneficial microorganism (usually *Pseudomonas*) that are able to induce ISR [21,73–79]. Still, just a few have described the molecular changes after the challenge with a pathogen [74,80–82] and as far as we know, none of these studies report the molecular changes in the early stages of the priming response (i.e. 1 hpi).

As sessile organisms, plants have to adapt themselves, using their molecular armory, in order to tolerate or resist several kinds of environmental stresses. In these responses, it seems that the celerity to respond is the pivotal element between success and failure [83–85]. For instance, *P*. *phytofirmans* PsJN accelerates the reaction to salt stress from 24 to 1 hour in *Arabidopsis* plants, inducing the accumulation of protective osmolytes, regulating ABA and JA signaling pathways and affecting the transcription of specific ion transporters [33]. All these accelerated responses are associated with better performance and recovery under short-term or sustained salt-stress in plants [33].

We previously demonstrated that the same strain protects *Arabidopsis* plants from the virulent pathogen *P*. *syringae* DC3000 trough ISR [41]. Here, we found that strain PsJN regulates 405 genes 13 days after sowing, which is in agreement with the transcriptional changes reported by Poupin et al. [34] in the same plant model. These genes correspond to approximately 1.8% of the analyzed *Arabidopsis* transcriptome, and could partly explain the primed state in the strain PsJN-inoculated plants. The highest transcriptional changes occurred 1 hour after the challenge of these plants with the pathogen, where strain PsJN regulated 645 genes (1 hpi); later that regulation was reduced to 420 genes (24 hpi). Recently, Wang et al. [78] compared the molecular responses of *Arabidopsis* plants to the ISR-inducing strain, *Bacillus cereus* AR156 with those to flg22, a pathogen-associated molecular pattern able to induce plant immunity. They found that both treatments co-regulate a high number of genes, suggesting that the defense responses triggered by AR156 are similar to the basal defense responses triggered by this molecular pattern. Here, a PCA showed a clear separation between the plant responses to infection associated to a previous inoculation with PsJN (Fig 2), and only 13% and 29.7% of the DEGs were co-regulated by PsJN and *Pst* DC3000 at 1 and 24 hpi, respectively (Fig 4B). Concordantly, a PsJN mutant strain lacking its flagella (*PsJN ΔfliA*) is still able to significantly protect *Arabidopsis* plants from an infection with strain DC3000 [41]. In this context, two hypotheses arise, (1) strain PsJN is accelerating the same response that occurs later in non-inoculated plants (24 hpi), and basically the regulated genes are the same or (2), strain PsJN triggers a different plant response, in time and quality, when plants face the pathogen. Interestingly, when the DEGs are compared having *Pst DC3000* as the factor inside each group of strain PsJN- and non-inoculated plants (as shown in Fig 4), only 11 genes that were regulated by strain PsJN at 1 hpi were also regulated at 24 hpi in non-inoculated plants, supporting the second hypothesis (S3 Table, intersection). Of these eleven genes, two were up-regulated; the first one corresponds to *LEGUME LECTIN FAMILY PROTEIN (LEC)*, which is associated to the JA/ET signaling pathways [86]. The second gene was *AZI1*, which is involved in the plant immune defense priming not only under biotic stress (Systemic acquired resistance, SAR) [87] but also under abiotic stresses such as drought [88]. *AZI1* encodes for a lipid transfer protein (LTP)-like protein, belonging to the *EARLY ARABIDOPSIS ALUMINIUM-INDUCED GENE1 (EARLI1)* gene subfamily [89] and its role for long-distance signals related to SAR is the best documented so far [90]. Cecchini et al. [87] demonstrated that after inoculation of roots with the PGPR *P*. *fluorescens*, only the wild type plants but not the *azi1-1* mutants, showed a significant reduction of *P*. *syringae* growth in distal-infected leaves, indicating an essential role for this gene also in ISR. In our model, *AZI1* was up regulated as fast as at 1 hpi (fold change Log2: 2.22) in strain PsJN-inoculated plants, but this up-regulation was only observed at 24 hpi in non-inoculated plants (fold change Log2: 2.47) (S2 Table).

Recently, a central role for N-hydroxy-pipecolic acid (NHP) was proposed as a mobile signal for SAR in *Arabidopsis* [91,92]. FMO1, *FLAVIN-DEPENDENT MONOOXYGENASE 1*, can synthesize NHP from pipecolic acid *in planta* and is indispensable for SAR induction [93]. Plant FMOs are divided into three clades and clade I contains *FMO1* and a pseudogene [94]. In our study, no changes were detected in *FMO1* expression either with or without a previous inoculation with PsJN, which suggests that, at least at a transcriptional level, this pathway might not be involved in this ISR defense mechanism.

A role for the plant hormones SA, JA, and ET has been raised in ISR where, again, the predominance of each signaling pathway will strongly depend on the players that are interacting [95]. For instance, the PGPR *B*. *cereus* AR156 requires both the SA and JA/ET signaling pathways and NPR1 to activate ISR against the biotrophic pathogen *Pst* DC3000 [96], but only the JA/ET hormonal pathways to trigger ISR against the necrotrophic pathogen *B*. *cinerea* [13]. In our previous report [41], we established that *P*. *phytofirmans* PsJN induces systemic resistance to *Pst* DC3000 trough a mechanism dependent of SA, JA and ET, since protection is only abolished when a triple mutant of these pathways is used. In this work, we found that strain PsJN induced transcriptional changes related to these hormonal pathways even before the challenge with *Pst* DC3000 (Fig 3A), and that these changes (especially with ET) are increased at 1 hpi (Fig 3B). This is in agreement with Poupin et al. [35] where it was reported that the ET signaling pathway is crucial for the plant growth-promotion effects associated with strain PsJN in *Arabidopsis*.

### Network analysis identifies functional relationships associated with strain PsJN induced systemic resistance

In addition to hormones, plant defense responses require diverse types of TFs modulating the expression of a vast number of responding genes. A better understanding of how these TFs underlie ISR could be helpful to identify key molecular players in this important defense response. Additionally, as TFs within the same family are evolutionarily closely related, they are likely regulated by common mechanisms [97]. Then, the recognition of general features of the regulation of whole TFs’ families can provide useful clues to better characterize the function of these members in a specific plant response. TFs’ families such as bZIP, bHLH, ERF/AP2, MYB, MYC, NAC, and WRKY have been related to disease resistance [98]. Here, we report that strain PsJN regulates the expression of 48 TF-coding genes at 1 hpi (Table 1). One of the families that presented only up-regulated genes was WRKY. WRKY TFs are one of the largest families of transcriptional regulators found exclusively in plants; they can act as transcriptional activators or repressors and are related to defense responses, abiotic stress response and developmental-associated processes [99,100]. Regarding defense responses, WRKY proteins have been linked not only to SAR but also to ISR. For instance, WRKY70 and WRKY11 were reported as regulators in *B*. *cereus* AR156-induced systemic resistance to *P*. *syringae* DC3000 in *Arabidopsis* [101]. Here, we found that WRKY26, 27, 28 and 47 were up-regulated at 1 hpi in plants with induced systemic resistance, suggesting an important role of this family in the regulation of this ISR mechanism.

Allu et al. [102] suggested a role for ANAC32 in the response to strain DC3000 in *Arabidopsis*, where this TF would induce SA signaling, thus repressing JA responsive genes, such as *PDF1*.*2*. We determined that *ANAC32* is down regulated in strain PsJN-inoculated plants at 1 hpi, and more interestingly, this down-regulation started before the challenge (0 hpi) and remained at 24 hpi. Concordantly, *PDF1*.*2* showed an up-regulation 0 hpi as was also reported previously [34,41]. The GCC-box is a binding site for members of the AP2/ERF family of TFs [103], such as ERF1 and ORA59, which are both important activators of *PDF1*.*2* [104]. Van der Does et al. [105] reported that SA reduces ORA59 protein accumulation, and that overexpression of ORA59 negatively affects the ability of SA to suppress *PDF1*.*2* gene expression, giving ORA59 an important role in the outcome of the SA/JA antagonism. Similarly, Pangesti et al. [106] found that *Pseudomonas simiae* WCS417r (formerly *P*. *fluorescens*) mediated ISR in *Arabidopsis* against the herbivore *Mamestra brassicae* inducing ORA59, which favors the JA/ET signaling pathways over JA pathway regulated by MYC2. We described that *ORA59* was up regulated at 1 hpi in PsJN-inoculated plants, highlighting the importance of a specific cross-regulation between SA and JA in this defense process. Additionally, the Redox Responsive Transcription Factor1 (RRTF1/ERF109) is regulated by JA, and in *Arabidopsis* it is induced rapidly and transiently by H_2_O_2_, as well as by biotic- and abiotic-induced redox signals [107]. In *Arabidopsis*, Matsuo et al. [107] showed that the inactivation and up-regulation of this gene reduces and increases reactive oxygen species (ROS) accumulation under stress, respectively. ROS act as a second messenger in stress response, but also their accumulation prompts to oxidation of several molecules and cell damage [108,109]. When ROS accumulate at later stages, or are formed as a consequence of mitochondrial damage, JA synthesis is over induced together with an autocatalytic oxidative burst [83]. We report here that RRTF1 was down regulated by strain PsJN under the challenge with *Pst* DC3000 at 1 hpi (Table 1). This correlates with the reduced superoxide anion accumulation that is observed in strain PsJN-inoculated plants after the infection with *Pst* DC3000 in comparison with the non-inoculated plants [41].

MYB72 has a significant role in ISR induced in *Arabidopsis* by the γ-proteobacterium *P*. *simiae* WCS417 [110], where this TF is involved in iron uptake and can modulate the root microbiome assembly [111]. In our study, *MYB72* was not transcriptionally affected in any of the treatments and measured times. Therefore, at least with our experimental strategy, a role for this regulator cannot be suggested in the systemic defense induced by this β-proteobacterium.

Network inference could be a valuable tool to uncover central hubs, gene regulatory sub-networks and functional relationships in a transcriptome response. Hence, with data from co-regulatory networks, it is possible to highlight novel clusters of plant genes contributing to the same biological processes or signal transduction pathways [112]. For instance, Weston et al. [76] described different plant co-expression in response to two *P*. *fluorescens* strains, finding that even though both strains co-regulated some genes; there was strain dependence in the network architectures. By means of system biology strategies, here we inferred a gene network of 500 interconnected genes that clustered in four co-expression modules or clusters, where LHY1, WRKY28, MYB31 and a *Redox-responsive* TF (RRTF1/ ERF109) were the most connected and central TFs (Fig 5). Of these, *RRTF1* and LHY were down regulated and *WRKY28* and *MYB31* were up regulated. Further analysis of the resulting clusters confirmed the biological identity of these sub-networks as pathways or processes controlled by ISR. For example, defense response, detoxification, induced systemic resistance, response to chitin, and oxidative stress were overexpressed in our network analysis, showing the importance of these processes in plant resistance triggered by strain PsJN. Moreover, the potential role of JA and ET was again evident from the gene network analysis. As mentioned before, these hormones are key for ISR induced by strain PsJN [41]. Interestingly, genes associated with SA and ABA responses were also regulated by the infection (1 hpi) in inoculated plants. These results support a model in which strain PsJN regulation of plant hormone response is complex and may allow the plant tolerance to several stress conditions, as shown in Pinedo et al. [33], where a role for ABA was proposed to salt-stress tolerance induction by strain PsJN in *Arabidopsis* plants.

This study showed that *WRKY28*, which is known to be up-regulated under drought and oxidative stress in *Arabidopsis* [113], was up-regulated at 1 hpi. Moreover, previous studies in plants overexpressing AtWRKY28 and AtWRKY75 suggested that these TFs are transcriptional regulators of SA and JA/ET-dependent defense signaling pathways in *Arabidopsis* [114]. Consistent with this idea, we found that several TFs, belonging to NAC and WRKY families and associated to SA and JA/ET pathways, grouped with WRK28, suggesting a regulatory sub-network based on these hormones. Within another cluster of the network, we found that MYB31 was also up regulated at 1 hpi. It has been described that this TF is induced in response to auxin [115]. LHY1 is a down-regulated TF predicted as a central regulator in cluster 3. Concordantly, APRR7, which is a transcriptional repressor of LHY [116] showed an up-regulation at 1 hpi. Given that LHY1 has a central role controlling the circadian rhythm [117,118], this gene and its targets may be integrators of internal metabolism and environmental conditions under PsJN induced resistance.

In summary, we found that strain PsJN induces a rapid and specific molecular response that is strongly related to jasmonate, ethylene, salicylic acid and ROS signaling pathways and that supports plant resistance to *P*. *syringae*. Additionally, the network analysis suggests a mechanism for both transcriptional induction and repression of TFs, with a highly coordinated regulation and potential roles for specific TF as hubs controlling the priming effect of *P*. *phytofirmans* PsJN in *Arabidopsis*, in the context of a biotic stress.

## Supporting information

S1 Fig Gene Ontology (GO) terms enriched in up- and down-regulated genes in *Paraburkholderia phytofirmans* PsJN-inoculated plants one hour after *Pseudomonas syringae* DC3000 infection. The VirtualPlant platform [1] was utilized in order to determine which GO terms were statistically overrepresented in comparison with the GO term represented in the *Arabidopsis* genome arrays (Fisher Exact Test with FDR correction, *p*<0.01). Bars denote the proportion of differentially expressed (DEGs) genes in each term relative to the total number of DEGs up- (A, blue) or down-regulated (B, orange) in strain PsJN-inoculated plants one hour after *Pst* DC3000 infection, in comparison to control plants (without strain PsJN inoculation) equivalently infected with *Pst DC3000*. To avoid redundancy, significant GO term lists were reduced using REVIGO [2], but full lists of significant GO terms, including differentially expressed genes that map to each term, are presented in S3 Table.
(PDF)

S1 Table Complete list of *Arabidopsis* genes regulated by *Paraburkholderia phytofirman*s PsJN before and after the challenge with *Pseudomonas syringae* pv. tomato DC3000. (XLSX)

S2 Table Full lists of significant Gene Ontology (GO) terms enriched in up- and down-regulated genes in *Paraburkholderia phytofirmans* PsJN-inoculated plants one hour after *Pseudomonas syringae* pv. tomato DC3000 infection, including differentially expressed genes that map to each term. (XLSX)

S3 Table Complete list of genes regulated by *Pseudomonas syringae* pv. tomato DC3000 in *Arabidopsis* plants that were inoculated or non-inoculated with *Paraburkholderia phytofirmans* PsJN. (XLSX)

## References

[1] Oerke E-C. Crop losses to pests. J Agric Sci. 2006;144: 31 10.1017/S0021859605005708

[2] Esker P, Savary S, McRoberts N. Crop loss analysis and global food supply: focusing now on required harvests. CAB Rev Perspect Agric Vet Sci Nutr Nat Resour. 2012;7: 52 10.1079/PAVSNNR20127052

[3] Sparks TC, Lorsbach BA. Perspectives on the agrochemical industry and agrochemical discovery. Pest Manag Sci. 2017;73: 672–677. 10.1002/ps.4457 27753242

[4] Windram O, Penfold CA, Denby KJ. Network modeling to understand plant immunity. Annu Rev Phytopathol. 2014;52: 93–111. 10.1146/annurev-phyto-102313-050103 24821185

[5] Thomma BP, Penninckx IA, Broekaert WF, Cammue BP. The complexity of disease signaling in *Arabidopsis*. Curr Opin Immunol. 2001;13: 63–8. 10.1016/S0952-7915(00)00183-7 11154919

[6] Glazebrook J. Contrasting mechanisms of defense against biotrophic and necrotrophic pathogens. Annu Rev Phytopathol. 2005;43: 205–227. 10.1146/annurev.phyto.43.040204.135923 16078883

[7] Jones JDG, Dangl JL. The plant immune system. Nature. 2006;444: 323–329. 10.1038/nature05286 17108957

[8] Pieterse CMJ, Leon-Reyes A, Van der Ent S, Van Wees SCM. Networking by small-molecule hormones in plant immunity. Nat Chem Biol. 2009;5: 308–316. 10.1038/nchembio.164 19377457

[9] Greenberg JT, Van Wees SCM, Caarls L, Pieterse CMJ. How salicylic acid takes transcriptional control over jasmonic acid signaling. Front Plant Sci. 2015;6: 170 10.3389/fpls.2015.00170 25859250 PMC4373269

[10] Verma V, Ravindran P, Kumar PP. Plant hormone-mediated regulation of stress responses. BMC Plant Biol. 2016;16: 86 10.1186/s12870-016-0771-y 27079791 PMC4831116

[11] Vlot AC, Dempsey DA, Klessig DF. Salicylic acid, a multifaceted hormone to combat disease. Annu Rev Phytopathol. 2009;47: 177–206. 10.1146/annurev.phyto.050908.135202 19400653

[12] Farmer EE, Alméras E, Krishnamurthy V. Jasmonates and related oxylipins in plant responses to pathogenesis and herbivory. Curr Opin Plant Biol. 2003;6: 372–378. 10.1016/S1369-5266(03)00045-1 12873533

[13] Nie P, Li X, Wang S, Guo J, Zhao H, Niu D. Induced systemic resistance against *Botrytis cinerea* by Bacillus cereus AR156 through a JA/ET- and NPR1-dependent signaling pathway and activates PAMP-triggered immunity in *Arabidopsis*. Front Plant Sci. 2017;8: 238 10.3389/fpls.2017.00238 28293243 PMC5329000

[14] Van der Ent S, Van Hulten M, Pozo M, Czechowski T, Udvardi M, Pieterse C, et al Priming of plant innate immunity by rhizobacteria and beta-aminobutyric acid: differences and similarities in regulation. New Phytol. 2009;183: 419–31. 10.1111/j.1469-8137.2009.02851.x 19413686

[15] Van Loon LC. Plant responses to plant growth-promoting rhizobacteria. Eur J Plant Pathol. 2007;119: 243–254. 10.1007/s10658-007-9165-1

[16] Van Loon LC, Bakker P, Pieterse C. Systemic resistance induced by rhizosphere bacteria. Annu Rev Phytopathol. 1998;36: 453–483. 10.1146/annurev.phyto.36.1.453 15012509

[17] Naznin HA, Kiyohara D, Kimura M, Miyazawa M, Shimizu M, Hyakumachi M. Systemic resistance induced by volatile organic compounds emitted by plant growth-promoting fungi in Arabidopsis thaliana. PLoS One. 2014;9: e86882 10.1371/journal.pone.0086882 24475190 PMC3903595

[18] Balmer A, Pastor V, Gamir J, Flors V, Mauch-Mani B. The “prime-ome”: towards a holistic approach to priming. Trends Plant Sci. 2015;20: 443–452. 10.1016/j.tplants.2015.04.002 25921921

[19] Van Hulten M, Pelser M, Van Loon LC, Pieterse C, Ton J. Costs and benefits of priming for defense in *Arabidopsis*. Proc Natl Acad Sci. 2006;103: 5602–5607. 10.1073/pnas.0510213103 16565218 PMC1459400

[20] Bakker PAHM, Pieterse CMJ, de Jonge R, Berendsen RL. The soil-borne legacy. Cell. 2018;172: 1178–1180. 10.1016/j.cell.2018.02.024 29522740

[21] Stringlis IA, Proietti S, Hickman R, Van Verk MC, Zamioudis C, Pieterse CMJ. Root transcriptional dynamics induced by beneficial rhizobacteria and microbial immune elicitors reveal signatures of adaptation to mutualists. Plant J. 2018;93: 166–180. 10.1111/tpj.13741 29024173 PMC5765484

[22] Beneduzi A, Ambrosini A, Passaglia L. Plant growth-promoting rhizobacteria (PGPR): Their potential as antagonists and biocontrol agents. Genet Mol Biol. 2012;35: 1044–1051. 23411488 10.1590/s1415-47572012000600020 PMC3571425

[23] Bhattacharyya PN, Jha DK. Plant growth-promoting rhizobacteria (PGPR): emergence in agriculture. World J Microbiol Biotechnol. 2012;28: 1327–50. 10.1007/s11274-011-0979-9 22805914

[24] Babalola O. Beneficial bacteria of agricultural importance. Biotechnol Lett. 2010;32: 1559–70. 10.1007/s10529-010-0347-0 20635120

[25] Sawana A, Adeolu M, Gupta RS. Molecular signatures and phylogenomic analysis of the genus *Burkholderia*: Proposal for division of this genus into the emended genus *Burkholderia* containing pathogenic organisms and a new genus *Paraburkholderia* gen. nov. harboring environmental species. Front Genet. 2014;5: 1–22. 10.3389/fgene.2014.00001 25566316 PMC4271702

[26] Barka EA, Nowak J, Clément C. Enhancement of chilling resistance of inoculated grapevine plantlets with a plant growth-promoting rhizobacterium, *Burkholderia phytofirmans* strain PsJN. Appl Environ Microbiol. 2006;72: 7246–7252. 10.1128/AEM.01047-06 16980419 PMC1636148

[27] Issa A, Esmaeel Q, Sanchez L, Courteaux B, Guise J-F, Gibon Y, et al Impacts of *Paraburkholderia phytofirmans* strain PsJN on tomato (*Lycopersicon esculentum* L.) under high temperature. Front Plant Sci. 2018;9: 1397 10.3389/fpls.2018.01397 30405648 PMC6201190

[28] Kurepin L, Park J, Lazarovits G, Bernards M. *Burkholderia phytofirmans*-induced shoot and root growth promotion is associated with endogenous changes in plant growth hormone levels. Plant Growth Regul. 2015;75: 199–207. 10.1007/s10725-014-9944-6

[29] Sessitsch A, Coenye T, Sturz A V., Vandamme P, Barka E, Salles JF, et al *Burkholderia phytofirmans* sp. nov., a novel plant-associated bacterium with plant-beneficial properties. Int J Syst Evol Microbiol. 2005;55: 1187–1192. 10.1099/ijs.0.63149-0 15879253

[30] Frommel MI, Nowak J, Lazarovits G. Growth enhancement and developmental modifications of *in vitro* grown potato (*Solanum tuberosum* spp. *tuberosum*) as affected by a nonfluorescent *Pseudomonas* sp. Plant Physiol. 1991;96: 928–936. 10.1104/pp.96.3.928 16668277 PMC1080867

[31] Miotto-Vilanova L, Jacquard C, Courteaux B, Wortham L, Michel J, Clément C, et al *Burkholderia phytofirmans* PsJN confers grapevine resistance against *Botrytis cinerea* via a direct antimicrobial effect combined with a better resource mobilization. Front Plant Sci. 2016;7: 1236 10.3389/fpls.2016.01236 27602036 PMC4993772

[32] Wang B, Mei C, Seiler J. Early growth promotion and leaf level physiology changes in *Burkholderia phytofirmans* strain PsJN inoculated switchgrass. Plant Physiol Biochem. 2015;86: 16–23. 10.1016/j.plaphy.2014.11.008 25461696

[33] Pinedo I, Ledger T, Greve M, Poupin MJ. *Burkholderia phytofirmans* PsJN induces long-term metabolic and transcriptional changes involved in *Arabidopsis thalian*a salt tolerance. Front Plant Sci. 2015; 10.3389/fpls.2015.00466 26157451 PMC4477060

[34] Poupin MJ, Timmermann T, Vega A, Zúñiga A, González B. Effects of the plant growth-promoting bacterium *Burkholderia phytofirmans* PsJN throughout the life cycle of *Arabidopsis thaliana*. PLoS One. 2013;8: 22–24. 10.1371/journal.pone.0069435 23869243 PMC3711820

[35] Poupin MJ, Greve M, Carmona V, Pinedo I. A complex molecular interplay of auxin and ethylene signaling pathways is involved in *Arabidopsis* growth promotion by *Burkholderia phytofirmans* PsJN. Front Plant Sci. 2016; 10.3389/fpls.2016.00492 27148317 PMC4828629

[36] Fernandez O, Vandesteene L, Feil R, Baillieul F, Lunn JE, Clément C. Trehalose metabolism is activated upon chilling in grapevine and might participate in *Burkholderia phytofirmans* induced chilling tolerance. Planta. 2012;236: 355–369. 10.1007/s00425-012-1611-4 22367062

[37] Ledger T, Rojas S, Timmermann T, Pinedo I, Poupin MJ, Garrido T, et al Volatile-mediated effects predominate in *Paraburkholderia phytofirmans* growth promotion and salt stress tolerance of *Arabidopsis thaliana*. Front Microbiol. 2016;7: 1–18. 10.3389/fmicb.2016.00001 27909432 PMC5112238

[38] Naveed M, Mitter B, Reichenauer TG, Wieczorek K, Sessitsch A. Increased drought stress resilience of maize through endophytic colonization by *Burkholderia phytofirmans* PsJN and *Enterobacter* sp. FD17. Environ Exp Bot. 2014;97: 30–39. 10.1016/j.envexpbot.2013.09.014

[39] Theocharis A, Bordiec S, Fernandez O, Paquis S, Dhondt-Cordelier S, Baillieul F, et al *Burkholderia phytofirmans* PsJN primes *Vitis vinifera* L. and confers a better tolerance to low nonfreezing temperatures. Mol Plant-Microbe Interact. 2012;25: 241–249. 10.1094/MPMI-05-11-0124 21942451

[40] Su F, Jacquard C, Villaume S, Michel J, Rabenoelina F, Clément C, et al *Burkholderia phytofirmans* PsJN reduces impact of freezing temperatures on photosynthesis in *Arabidopsis thaliana*. Front Plant Sci. 2015;6: 1–13. 10.3389/fpls.2015.00001 26483823 PMC4591482

[41] Timmermann T, Armijo G, Donoso R, Seguel A, Holuigue L, González B. Paraburkholderia phytofirmans PsJN protects Arabidopsis thaliana against a virulent strain of Pseudomonas syringae through the activation of induced resistance. Mol Plant-Microbe Interact. 2017;30: 215–230. 10.1094/MPMI-09-16-0192-R 28118091

[42] Dorn E, Hellwig M, Reineke W, Knackmus HJ. Isolation and characterization of a 3-chlorobenzoate degrading *pseudomonad*. Arch Microbiol. 1974;99: 61–70. 10.1007/BF00696222 4852581

[43] Murashige T, Skoog F. A revised medium for rapid growth and bio assays with tobacco tissue cultures. Physiol Plant. 1962;15: 473–497. 10.1111/j.1399-3054.1962.tb08052.x

[44] Boyes D, Zayed A, Ascenzi R, McCaskill A, Hoffman N, Davis K, et al Growth stage-based phenotypic analysis of *Arabidopsis*: a model for high throughput functional genomics in plants. Plant Cell. 2001;13: 1499–1510. 10.1105/TPC.010011 11449047 PMC139543

[45] Irizarry RA, Hobbs B, Collin F, Beazer‐Barclay YD, Antonellis KJ, Scherf U, et al Exploration, normalization, and summaries of high density oligonucleotide array probe level data. Biostatistics. 2003;4: 249–264. 10.1093/biostatistics/4.2.249 12925520

[46] Gautier L, Cope L, Bolstad BM, Irizarry RA. affy—analysis of Affymetrix GeneChip data at the probe level. Bioinformatics. 2004;20: 307–315. 10.1093/bioinformatics/btg405 14960456

[47] Edgar R, Domrachev M, Lash AE. Gene Expression Omnibus: NCBI gene expression and hybridization array data repository. Nucleic Acids Res. 2002;30: 207–10. 10.1093/nar/30.1.207_5 11752295 PMC99122

[48] Benjamini Y, Hochberg Y. Controlling the false discovery rate: A practical and powerful approach to multiple testing. J R Stat Soc Ser B. 1995;57: 289–300. 10.2307/2346101

[49] Saeed AI, Sharov V, White J, Li J, Liang W, Bhagabati N, et al TM4: A free, open-source system for microarray data management and analysis. Biotechniques. 2003;34: 374–378. 10.2144/03342mt01 12613259

[50] Breitling R, Armengaud P, Amtmann A, Herzyk P. Rank products: a simple, yet powerful, new method to detect differentially regulated genes in replicated microarray experiments. FEBS Lett. 2004;573: 83–92. 10.1016/j.febslet.2004.07.055 15327980

[51] Raychaudhuri S, Stuart JM, Altman RB. Principal components analysis to summarize microarray experiments: application to sporulation time series. Pacific Symp Biocomput. 2000; 455–66. 10.1142/9789814447331_0043 PMC2669932 10902193

[52] Katari MS, Nowicki SD, Aceituno FF, Nero D, Kelfer J, Parnell Thompson L, et al Bioinformatics VirtualPlant: A software platform to support systems biology research. Plant Physiol. 2010;152: 500–515. 10.1104/pp.109.147025 20007449 PMC2815851

[53] Supek F, Bošnjak M, Škunca N, Šmuc T. REVIGO summarizes and visualizes long lists of gene ontology terms. PLoS One. 2011;6: e21800 10.1371/journal.pone.0021800 21789182 PMC3138752

[54] Thimm O, Bläsing O, Gibon Y, Nagel A, Meyer S, Krüger P, et al MAPMAN: a user-driven tool to display genomics data sets onto diagrams of metabolic pathways and other biological processes. Plant J. 2004;37: 914–39. 10.1111/j.1365-313X.2004.02016.x 14996223

[55] Jin J, Tian F, Yang D-C, Meng Y-Q, Kong L, Luo J, et al PlantTFDB 4.0: toward a central hub for transcription factors and regulatory interactions in plants. Nucleic Acids Res. 2017;45: D1040–D1045. 10.1093/nar/gkw982 27924042 PMC5210657

[56] Pérez-Rodríguez P, Riaño-Pachón DM, Corrêa LGG, Rensing SA, Kersten B, Mueller-Roeber B. PlnTFDB: updated content and new features of the plant transcription factor database. Nucleic Acids Res. 2010;38: D822–7. 10.1093/nar/gkp805 19858103 PMC2808933

[57] Aoki Y, Okamura Y, Tadaka S, Kinoshita K, Obayashi T. ATTED-II in 2016: a plant coexpression database towards lineage-specific coexpression. Plant Cell Physiol. 2016;57: e5–e5. 10.1093/pcp/pcv165 26546318 PMC4722172

[58] Obayashi T, Okamura Y, Ito S, Tadaka S, Aoki Y, Shirota M, et al ATTED-II in 2014: evaluation of gene coexpression in agriculturally important plants. Plant Cell Physiol. 2014;55: e6 10.1093/pcp/pct178 24334350 PMC3894708

[59] O’Malley RC, Huang S-SC, Song L, Lewsey MG, Bartlett A, Nery JR, et al Cistrome and epicistrome features shape the regulatory DNA landscape. Cell. 2016;165: 1280–1292. 10.1016/j.cell.2016.04.038 27203113 PMC4907330

[60] Shannon P, Markiel A, Ozier O, Baliga NS, Wang JT, Ramage D, et al Cytoscape: a software environment for integrated models of biomolecular interaction networks. Genome Res. 2003;13: 2498–504. 10.1101/gr.1239303 14597658 PMC403769

[61] Morris JH, Apeltsin L, Newman AM, Baumbach J, Wittkop T, Su G, et al clusterMaker: a multi-algorithm clustering plugin for Cytoscape. BMC Bioinformatics. 2011;12: 436 10.1186/1471-2105-12-436 22070249 PMC3262844

[62] Su G, Kuchinsky A, Morris JH, States DJ, Meng F. GLay: community structure analysis of biological networks. Bioinformatics. 2010;26: 3135–7. 10.1093/bioinformatics/btq596 21123224 PMC2995124

[63] Bindea G, Mlecnik B, Hackl H, Charoentong P, Tosolini M, Kirilovsky A, et al ClueGO: a Cytoscape plug-in to decipher functionally grouped gene ontology and pathway annotation networks. Bioinformatics. 2009;25: 1091–1093. 10.1093/bioinformatics/btp101 19237447 PMC2666812

[64] Czechowski T, Stitt M, Altmann T, Udvardi MK, Scheible W-R. Genome-wide identification and testing of superior reference genes for transcript normalization in *Arabidopsis*. Plant Physiol. 2005;139: 5–17. 10.1104/pp.105.063743 16166256 PMC1203353

[65] Poupin MJ, Federici F, Medina C, Matus JT, Timmermann T, Arce-Johnson P. Isolation of the three grape sub-lineages of B-class MADS-box TM6, PISTILLATA and APETALA3 genes which are differentially expressed during flower and fruit development. Gene. 2007;404: 10–24. 10.1016/j.gene.2007.08.005 17920788

[66] Bustin SA, Benes V, Garson JA, Hellemans J, Huggett J, Kubista M, et al The MIQE guidelines: Minimum information for publication of quantitative real-time PCR experiments. Clin Chem. 2009;55: 611–622. 10.1373/clinchem.2008.112797 19246619

[67] Sasaki-Sekimoto Y, Jikumaru Y, Obayashi T, Saito H, Masuda S, Kamiya Y, et al Basic helix-loop-helix transcription factors JASMONATE-ASSOCIATED MYC2-LIKE1 (JAM1), JAM2, and JAM3 are negative regulators of jasmonate responses in *Arabidopsis*. Plant Physiol. 2013;163: 291–304. 10.1104/pp.113.220129 23852442 PMC3762649

[68] Li Z, Peng J, Wen X, Guo H. Ethylene-insensitive3 is a senescence-associated gene that accelerates age-dependent leaf senescence by directly repressing miR164 transcription in *Arabidopsis*. Plant Cell. 2013;25: 3311–28. 10.1105/tpc.113.113340 24064769 PMC3809534

[69] Kim HJ, Hong SH, Kim YW, Lee IH, Jun JH, Phee B-K, et al Gene regulatory cascade of senescence-associated NAC transcription factors activated by ETHYLENE-INSENSITIVE2-mediated leaf senescence signalling in *Arabidopsis*. J Exp Bot. 2014;65: 4023–4036. 10.1093/jxb/eru112 24659488 PMC4106440

[70] Zhang X, Ivanova A, Vandepoele K, Radomiljac J, Van de Velde J, Berkowitz O, et al The transcription factor MYB29 is a regulator of ALTERNATIVE OXIDASE1a. Plant Physiol. 2017;173: 1824–1843. 10.1104/pp.16.01494 28167700 PMC5338668

[71] Gust AA, Biswas R, Lenz HD, Rauhut T, Ranf S, Kemmerling B, et al Bacteria-derived peptidoglycans constitute pathogen-associated molecular patterns triggering innate immunity in *Arabidopsis*. J Biol Chem. 2007;282: 32338–48. 10.1074/jbc.M704886200 17761682

[72] Zamioudis C, Hanson J, Pieterse CMJ. β-Glucosidase BGLU42 is a MYB72-dependent key regulator of rhizobacteria-induced systemic resistance and modulates iron deficiency responses in *Arabidopsis* roots. New Phytol. 2014;204: 368–379. 10.1111/nph.12980 25138267

[73] Wang Y, Ohara Y, Nakayashiki H, Tosa Y, Mayama S. Microarray analysis of the gene expression profile induced by the endophytic plant growth-promoting rhizobacteria, *Pseudomonas fluorescens* FPT9601-T5 in *Arabidopsis*. Mol Plant-Microbe Interact. 2005;18: 385–396. 10.1094/MPMI-18-0385 15915637

[74] Cartieaux F, Contesto C, Gallou A, Desbrosses G, Kopka J, Taconnat L, et al Simultaneous interaction of *Arabidopsis thaliana* with *Bradyrhizobium* sp. strain ORS278 and *Pseudomonas syringae* pv. tomato DC3000 leads to complex transcriptome changes. Mol Plant-Microbe Interact. 2008;21: 244–259. 10.1094/MPMI-21-2-0244 18184068

[75] Van de Mortel JE, de Vos RCH, Dekkers E, Pineda A, Guillod L, Bouwmeester K, et al Metabolic and transcriptomic changes induced in *Arabidopsis* by the rhizobacterium *Pseudomonas fluorescens* SS101. Plant Physiol. 2012;160: 2173–88. 10.1104/pp.112.207324 23073694 PMC3510139

[76] Weston DJ, Pelletier DA, Morrell-Falvey JL, Tschaplinski TJ, Jawdy SS, Lu T-Y, et al *Pseudomonas fluorescens* induces strain-dependent and strain-independent host plant responses in defense networks, primary metabolism, photosynthesis, and fitness. Mol Plant-Microbe Interact. 2012;25: 765–778. 10.1094/MPMI-09-11-0253 22375709

[77] Cheng X, Etalo DW, van de Mortel JE, Dekkers E, Nguyen L, Medema MH, et al Genome-wide analysis of bacterial determinants of plant growth promotion and induced systemic resistance by *Pseudomonas fluorescens*. Environ Microbiol. 2017;19: 4638–4656. 10.1111/1462-2920.13927 28892231

[78] Wang S, Zheng Y, Gu C, He C, Yang M, Zhang X, et al *Bacillus cereus* AR156 activates defense responses to *Pseudomonas syringae* pv. tomato in *Arabidopsis thaliana* similarly to flg22. Mol Plant-Microbe Interact. 2018;31: 311–322. 10.1094/MPMI-10-17-0240-R 29090631

[79] Mathys J, De Cremer K, Timmermans P, Van Kerckhove S, Lievens B, Vanhaecke M, et al Genome-wide characterization of ISR induced in *Arabidopsis thaliana* by *Trichoderma hamatum* T382 against *Botrytis cinerea* infection. Front Plant Sci. 2012;3: 108 10.3389/fpls.2012.00108 22661981 PMC3362084

[80] Duke KA, Becker MG, Girard IJ, Millar JL, Dilantha Fernando WG, Belmonte MF, et al The biocontrol agent Pseudomonas chlororaphis PA23 primes *Brassica napus* defenses through distinct gene networks. BMC Genomics. 2017;18: 467 10.1186/s12864-017-3848-6 28629321 PMC5477169

[81] Niu D, Wang X, Wang Y, Song X, Wang J, Guo J, et al *Bacillus cereus* AR156 activates PAMP-triggered immunity and induces a systemic acquired resistance through a NPR1-and SA-dependent signaling pathway. Biochem Biophys Res Commun. 2016;469: 120–125. 10.1016/j.bbrc.2015.11.081 26616055

[82] Verhagen BWM, Glazebrook J, Zhu T, Chang H-S, van Loon LC, Pieterse CMJ. The transcriptome of rhizobacteria-induced systemic resistance in *Arabidopsis*. Mol Plant-Microbe Interact. 2004;17: 895–908. 10.1094/MPMI.2004.17.8.895 15305611

[83] Ismail A, Takeda S, Nick P. Life and death under salt stress: same players, different timing? J Exp Bot. 2014;65: 2963–2979. 10.1093/jxb/eru159 24755280

[84] Julkowska MM, Testerink C. Tuning plant signaling and growth to survive salt. Trends Plant Sci. 2015;20: 586–594. 10.1016/j.tplants.2015.06.008 26205171

[85] Janiak A, Kwasniewski M, Sowa M, Gajek K, Żmuda K, Kościelniak J, et al No time to waste: Transcriptome study reveals that drought tolerance in barley may be attributed to stressed-like expression patterns that exist before the occurrence of stress. Front Plant Sci. 2017;8: 2212 10.3389/fpls.2017.02212 29375595 PMC5767312

[86] Lyou SH, Park HJ, Jung C, Sohn HB, Lee G, Kim CH, et al The *Arabidopsis* AtLEC gene encoding a lectin-like protein is up-regulated by multiple stimuli including developmental signal, wounding, jasmonate, ethylene, and chitin elicitor. Mol Cells. 2009;27: 75–81. 10.1007/s10059-009-0007-1 19214436

[87] Cecchini NM, Steffes K, Schläppi MR, Gifford AN, Greenberg JT. *Arabidopsis* AZI1 family proteins mediate signal mobilization for systemic defence priming. Nat Commun. 2015;6: 7658 10.1038/ncomms8658 26203923

[88] Atkinson NJ, Lilley CJ, Urwin PE. Identification of genes involved in the response of *Arabidopsis* to simultaneous biotic and abiotic stresses. Plant Physiol. 2013;162: 2028–2041. 10.1104/pp.113.222372 23800991 PMC3729780

[89] Richards KD, Schott EJ, Sharma YK, Davis KR, Gardner RC. Aluminum induces oxidative stress genes in *Arabidopsis thaliana*. Plant Physiol. 1998;116: 409–18. 10.1104/pp.116.1.409 9449849 PMC35183

[90] Bouain N, Satbhai SB, Korte A, Saenchai C, Desbrosses G, Berthomieu P, et al Natural allelic variation of the AZI1 gene controls root growth under zinc-limiting condition. Bomblies K, editor. PLOS Genet. 2018;14: e1007304 10.1371/journal.pgen.1007304 29608565 PMC5897037

[91] Chen Y-C, Holmes EC, Rajniak J, Kim J-G, Tang S, Fischer CR, et al N-hydroxy-pipecolic acid is a mobile metabolite that induces systemic disease resistance in *Arabidopsis*. Proc Natl Acad Sci; 2018;115: E4920–E4929. 10.1073/pnas.1805291115 29735713 PMC6003486

[92] Hartmann M, Zeier T, Bernsdorff F, Reichel-Deland V, Kim D, Hohmann M, et al Flavin monooxygenase-generated N-hydroxypipecolic acid is a critical element of plant systemic immunity. Cell. 2018;173: 456–469.e16. 10.1016/j.cell.2018.02.049 29576453

[93] Mishina TE, Zeier J. The *Arabidopsis* flavin-dependent monooxygenase FMO1 is an essential component of biologically induced systemic acquired resistance. Plant Physiol. 2006;141: 1666–75. 10.1104/pp.106.081257 16778014 PMC1533925

[94] Schlaich NL. Flavin-containing monooxygenases in plants: looking beyond detox. Trends Plant Sci.; 2007;12: 412–418. 10.1016/j.tplants.2007.08.009 17765596

[95] Wu G, Liu Y, Xu Y, Zhang G, Shen Q, Zhang R. Exploring elicitors of the beneficial rhizobacterium *Bacillus amyloliquefaciens* SQR9 to induce plant systemic resistance and their interactions with plant signaling pathways. Mol Plant-Microbe Interact. 2018; 31:560–567. 10.1094/MPMI-11-17-0273-R 29309236

[96] Niu D-D, Liu H-X, Jiang C-H, Wang Y-P, Wang Q-Y, Jin H-L, et al The plant growth–promoting rhizobacterium *Bacillus cereus* AR156 induces systemic resistance in *Arabidopsis thaliana* by simultaneously activating salicylate- and jasmonate/ethylene-dependent signaling pathways. Mol Plant-Microbe Interact. 2011;24: 533–542. 10.1094/MPMI-09-10-0213 21198361

[97] Llorca CM, Potschin M, Zentgraf U. bZIPs and WRKYs: two large transcription factor families executing two different functional strategies. Front Plant Sci. 2014;5: 169 10.3389/fpls.2014.00169 24817872 PMC4012195

[98] Singh KB, Foley RC, Oñate-Sánchez L. Transcription factors in plant defense and stress responses. Curr Opin Plant Biol. 2002;5: 430–436. 10.1016/S1369-5266(02)00289-3 12183182

[99] Bakshi M, Oelmüller R. WRKY transcription factors: Jack of many trades in plants. Plant Signal Behav. 2014;9: e27700 10.4161/psb.27700 24492469 PMC4091213

[100] Phukan UJ, Jeena GS, Shukla RK. WRKY transcription factors: Molecular regulation and stress responses in plants. Front Plant Sci. 2016;7: 760 10.3389/fpls.2016.00760 27375634 PMC4891567

[101] Jiang C-H, Huang Z-Y, Xie P, Gu C, Li K, Wang D-C, et al Transcription factors WRKY70 and WRKY11 served as regulators in rhizobacterium *Bacillus cereus* AR156-induced systemic resistance to *Pseudomonas syringae* pv. *tomato* DC3000 in *Arabidopsis*. J Exp Bot. 2016;67: 157–174. 10.1093/jxb/erv445 26433201

[102] Allu AD, Brotman Y, Xue G-P, Balazadeh S. Transcription factor ANAC032 modulates JA/SA signalling in response to *Pseudomonas syringae* infection. EMBO Rep. 2016;17: 1578–1589. 10.15252/embr.201642197 27632992 PMC5090710

[103] Hao D, Ohme-Takagi M, Sarai A. Unique mode of GCC box recognition by the DNA-binding domain of ethylene-responsive element-binding factor (ERF domain) in plant. J Biol Chem. 1998;273: 26857–61. 10.1074/jbc.273.41.26857 9756931

[104] Zarei A, Körbes AP, Younessi P, Montiel G, Champion A, Memelink J. Two GCC boxes and AP2/ERF-domain transcription factor ORA59 in jasmonate/ethylene-mediated activation of the PDF1.2 promoter in *Arabidopsis*. Plant Mol Biol. 2011;75: 321–31. 10.1007/s11103-010-9728-y 21246258 PMC3044237

[105] Van der Does D, Leon-Reyes A, Koornneef A, Van Verk MC, Rodenburg N, Pauwels L, et al Salicylic acid suppresses jasmonic acid signaling downstream of SCFCOI1-JAZ by targeting GCC promoter motifs via transcription factor ORA59. Plant Cell. 2013;25: 744–61. 10.1105/tpc.112.108548 23435661 PMC3608790

[106] Pangesti N, Reichelt M, van de Mortel JE, Kapsomenou E, Gershenzon J, van Loon JJA, et al Jasmonic acid and ethylene signaling pathways regulate glucosinolate levels in plants during rhizobacteria-induced systemic resistance against a leaf-chewing herbivore. J Chem Ecol. 2016;42: 1212–1225. 10.1007/s10886-016-0787-7 27848154 PMC5148788

[107] Matsuo M, Johnson JM, Hieno A, Tokizawa M, Nomoto M, Tada Y, et al High REDOX RESPONSIVE TRANSCRIPTION FACTOR1 levels result in accumulation of reactive oxygen species in *Arabidopsis thaliana* shoots and roots. Mol Plant. 2015;8: 1253–1273. 10.1016/j.molp.2015.03.011 25882345

[108] Apel K, Hirt H. Reactive oxygen species: Metabolism, oxidative stress, and signal transduction. Annu Rev Plant Biol. 2004;55: 373–399. 10.1146/annurev.arplant.55.031903.141701 15377225

[109] Golldack D, Li C, Mohan H, Probst N. Tolerance to drought and salt stress in plants: Unraveling the signaling networks. Front Plant Sci. 2014;5: 151 10.3389/fpls.2014.00151 24795738 PMC4001066

[110] Van der Ent S, Verhagen BWM, Van Doorn R, Bakker D, Verlaan MG, Pel MJC, et al MYB72 is required in early signaling steps of rhizobacteria-induced systemic resistance in *Arabidopsis*. Plant Physiol. 2008;146: 1293–304. 10.1104/pp.107.113829 18218967 PMC2259080

[111] Stringlis IA, Yu K, Feussner K, de Jonge R, Van Bentum S, Van Verk MC, et al MYB72-dependent coumarin exudation shapes root microbiome assembly to promote plant health. Proc Natl Acad Sci. 2018;115: E5213–E5222. 10.1073/pnas.1722335115 29686086 PMC5984513

[112] Berri S, Abbruscato P, Faivre-Rampant O, Brasileiro AC, Fumasoni I, Satoh K, et al Characterization of WRKY co-regulatory networks in rice and *Arabidopsis*. BMC Plant Biol. 2009;9: 120 10.1186/1471-2229-9-120 19772648 PMC2761919

[113] Babitha KC, Ramu S V., Pruthvi V, Mahesh P, Nataraja KN, Udayakumar M. Co-expression of AtbHLH17 and AtWRKY28 confers resistance to abiotic stress in *Arabidopsis*. Transgenic Res. 2013;22: 327–341. 10.1007/s11248-012-9645-8 22948308

[114] Chen X, Liu J, Lin G, Wang A, Wang Z, Lu G. Overexpression of AtWRKY28 and AtWRKY75 in *Arabidopsis* enhances resistance to oxalic acid and *Sclerotinia sclerotiorum*. Plant Cell Rep. 2013;32: 1589–1599. 10.1007/s00299-013-1469-3 23749099

[115] Goda H, Sawa S, Asami T, Fujioka S, Shimada Y, Yoshida S. Comprehensive comparison of auxin-regulated and brassinosteroid-regulated genes in *Arabidopsis*. Plant Physiol. 2004;134: 1555–73. 10.1104/pp.103.034736 15047898 PMC419831

[116] Huang W, Pérez-García P, Pokhilko A, Millar AJ, Antoshechkin I, Riechmann JL, et al Mapping the core of the *Arabidopsis* circadian clock defines the network structure of the oscillator. Science. 2012;336: 75–9. 10.1126/science.1219075 22403178

[117] Pruneda-Paz JL, Kay SA. An expanding universe of circadian networks in higher plants. Trends Plant Sci. 2010;15: 259–65. 10.1016/j.tplants.2010.03.003 20382065 PMC2866796

[118] Li Z, Bonaldi K, Uribe F, Pruneda-Paz JL. A localized *Pseudomonas syringae* infection triggers systemic clock responses in *Arabidopsis*. Curr Biol. 2018;28: 630–639.e4. 10.1016/j.cub.2018.01.001 29398214 PMC5820129

