# Supplementary figures and images for: Gene networks underlying the early regulation of Paraburkholderia phytofirmans PsJN induced systemic resistance in Arabidopsis
Source: PLoS One. 2019 Aug 22;14(8):e0221358. doi: 10.1371/journal.pone.0221358 (PMC6705864; doi:10.1371/journal.pone.0221358)

A

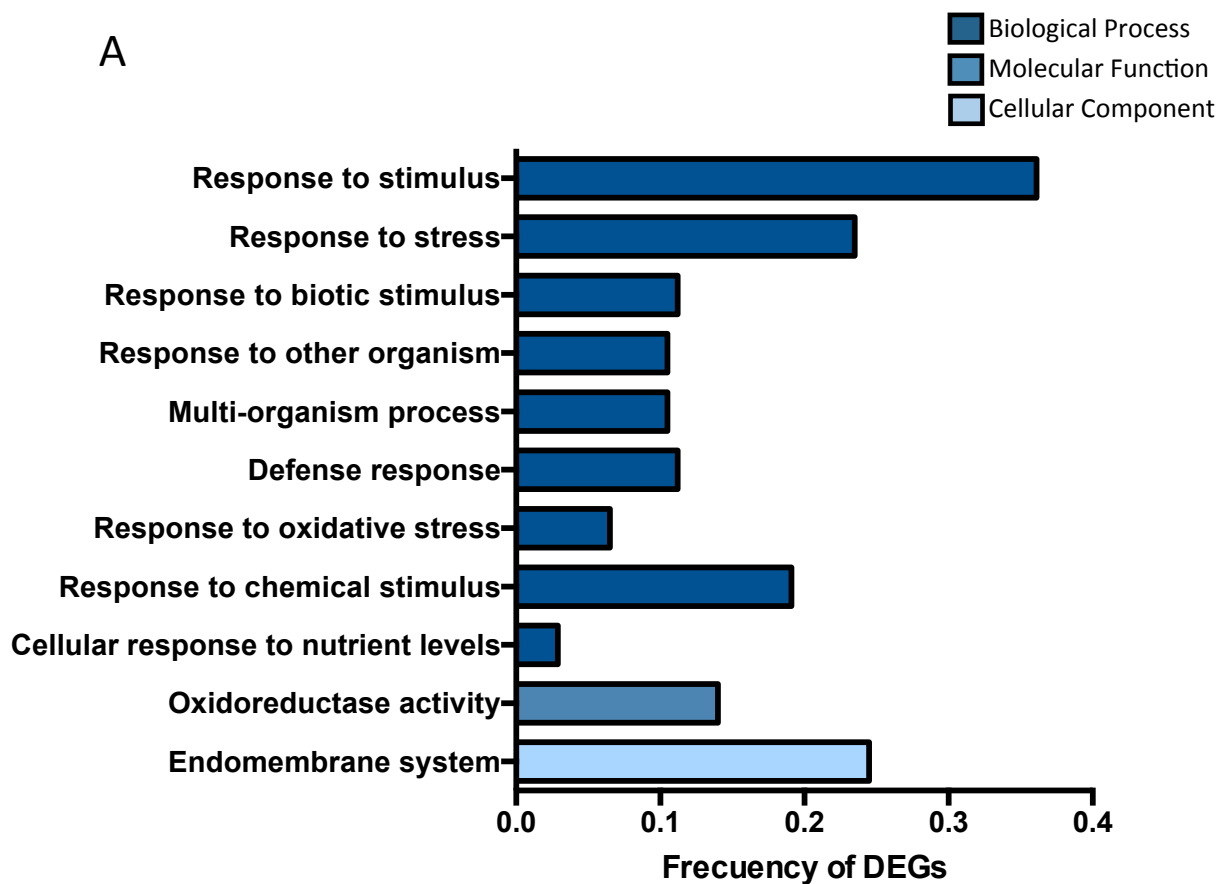

B

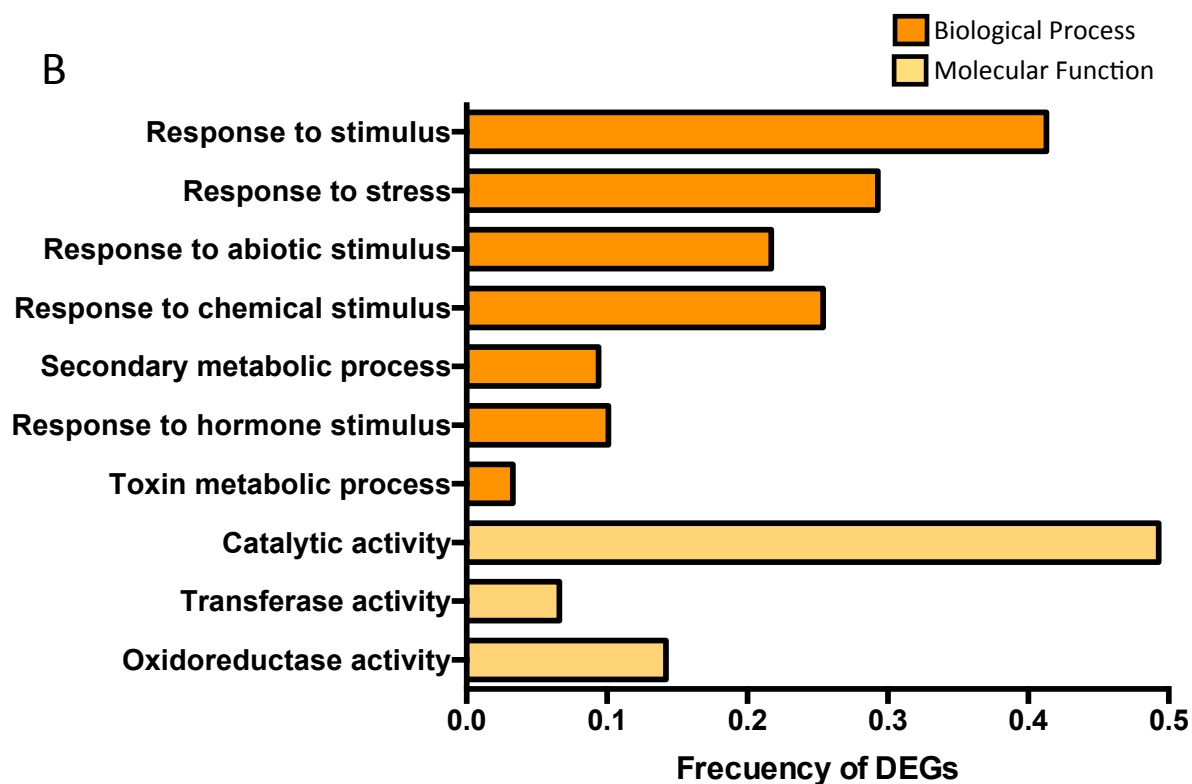

Supplement: S1 Fig — The VirtualPlant platform [1] was utilized in order to determine which GO terms were statistically overrepresented in comparison with the GO term represented in the Arabidopsis genome arrays (Fisher Exact Test with FDR correction, p<0.01). Bars denote the proportion of differentially expressed (DEGs) genes in each term relative to the total number of DEGs up- (A, blue) or down-regulated (B, orange) in strain PsJN-inoculated plants one hour after Pst DC3000 infection, in comparison to control plants (without strain PsJN inoculation) equivalently infected with Pst DC3000. To avoid redundancy, significant GO term lists were reduced using REVIGO [2], but full lists of significant GO terms, including differentially expressed genes that map to each term, are presented in S3 Table. (PDF) [file pone.0221358.s001.pdf]
